# Effects of digitalized traditional Chinese exercises on the physical and mental health and quality of life of older adults: a systematic review and meta-analysis of randomized controlled trials

**DOI:** 10.3389/fpubh.2025.1725847

**Published:** 2025-12-11

**Authors:** Yongjie Tu, Xiaoguang Lin, Jiongliang Zhang, Yujie Guan, Bin Zhao

**Affiliations:** 1 The Second Clinical College, Heilongjiang University of Chinese Medicine, Harbin, China; 2 Department of Musculoskeletal Pain, The Second Affiliated Hospital of Heilongjiang University of Chinese Medicine, Harbin, China

**Keywords:** meta-analysis, digital health, traditional Chinese exercises, healthy aging, older adult

## Abstract

**Objective:**

This study aims to systematically evaluate the effects of digitalized traditional Chinese exercises (TCEs) on physical function, cognitive function, mental health, and quality of life in older adults.

**Methods:**

Searches were conducted in the PubMed, Embase, Cochrane Library, Web of Science, CNKI, VIP, and Wan Fang databases from their inception until 28 September 2025 to identify relevant randomized controlled trials. Outcome measures included physical function, cognition, depression, and quality of life. The Cochrane Risk of Bias Assessment Tool and GRADE criteria were used to assess the quality of the studies. Statistical analyses were performed using RevMan 5.3 and Stata 17.0.

**Results:**

Sixteen trials involving 1,604 participants were included in this study. Low to moderate-certainty evidence showed that digitalized TCEs significantly improved functional mobility (WMD = −0.81; 95% CI, −1.28 to −0.33; *p* = 0.0009; I^2^ = 76%), balance function (SMD = 0.88; 95% CI, 0.68 to 1.08; *p* < 0.00001; I^2^ = 0%), and global cognition (WMD = 1.98; 95% CI, 1.44 to 2.52; *p* < 0.00001; I^2^ = 84%). In addition, moderate-certainty evidence indicated small but statistically significant improvements in quality of life (SMD = 0.17; 95% CI, 0.03 to 0.30; *p* = 0.01; I^2^ = 3%). However, digitalized TCEs did not significantly improve grip strength (WMD = −0.26; 95% CI, −1.15 to 0.62; *p* = 0.56; I^2^ = 0%; low certainty evidence) or depression (SMD = −0.71; 95% CI, −1.48 to 0.05; *p* = 0.07; I^2^ = 95%; very low certainty evidence).

**Conclusion:**

This study demonstrates that digitalized TCEs can significantly improve multiple health indicators in older adults, aligning with the WHO’s concept of healthy aging and offering a promising intervention to promote it. It is important to note that long-term participation is crucial for sustaining these health benefits; therefore, enhancing exercise adherence among older adults is essential.

**Systematic review registration:**

https://www.crd.york.ac.uk/PROSPERO/view/CRD420251053317, identifier CRD420251053317.

## 1 Introduction

Global population aging presents a significant challenge to public health systems worldwide. According to the World Health Organization (WHO), the population aged 60 years and older is projected to reach 2.1 billion by 2050 (1). This demographic shift is accompanied by an increasing burden of age-related functional decline and chronic diseases, as highlighted in the Global Burden of Disease study, with earlier onset and greater severity in low-resource areas (2). This burden significantly increases healthcare expenditures and imposes multiple health risks and sustained economic pressure on older adults (3, 4).

Over the past decade, numerous multidisciplinary interventions led by healthcare professionals have been developed to support active aging (5, 6). These interventions integrate exercise prescription, nutritional guidance, psychological support, and social activities, sometimes supplemented by pharmacological treatments (5, 6). While beneficial, these models face limitations, including reliance on professionals and inadequate coverage in rural areas (7). Moreover, research on healthy aging often emphasizes physical health while overlooking crucial dimensions, such as psychosocial factors (8).

The WHO emphasizes that healthy aging is a multidimensional process involving the maintenance of functional ability to ensure well-being. This process encompasses physical, cognitive, mental, and social domains, aiming to optimize quality of life while managing chronic conditions (9, 10). The ‘Active and Healthy Aging’ framework further highlights this holistic perspective (8). This approach is also reflected in research among Indigenous communities, where healthy aging is viewed as an integrated concept achieved through physical mobility, cognitive engagement, and social participation (11). However, this multidimensionality is not adequately captured in existing intervention studies or their syntheses.

Traditional Chinese exercises (TCEs), such as Tai Chi, Baduanjin, and Yijinjing, combine gentle movements, breath regulation, and mindfulness meditation to create a unique mind–body integration training method (12). Conventional TCEs, typically practiced under the guidance of an on-site instructor, have been shown to effectively improve physical function (13), mental health (14), cognitive function (15), and quality of life (16, 17). These benefits highlight the significant role of TCEs in promoting healthy aging. Nevertheless, their widespread adoption is hindered by challenges in limited time and space, complex and difficult-to-learn movements, and the lack of real-time guidance from professional instructors during home practice (18). Furthermore, the integration of exercise interventions into healthcare systems—referred to as ‘sport-medicine integration’—is impeded by a shortage of qualified professionals and a lack of prescribed exercise services (19).

Recent research has investigated the digital transformation of TCEs through various digital platforms, including pre-recorded instructional videos (20, 21), live video conferencing (22), virtual reality (VR) immersive systems (23, 24), motion capture games (25), and artificial intelligence (AI)-based movement correction applications (26). These studies have preliminarily demonstrated the feasibility and safety of digitalized TCEs. Such approaches enhance the effectiveness of home practice by increasing accessibility and flexibility, reducing the reliance of conventional TCEs on specific time and space constraints as well as professional guidance, thereby making them more suitable for a broader range of older adults. Although preliminary randomized controlled trials (RCTs) have demonstrated the synergistic effects of combining digital technology with TCEs, there remains a lack of systematic empirical research on integrated intervention models that merge the two. To date, no meta-analysis has specifically examined the impact of digitalized TCEs on the health of older adults.

To address this gap, this systematic review and meta-analysis will be the first to: (1) comprehensively evaluate the effects of digitalized TCEs on four core domains of older adults’ health—physical, cognitive, psychological functions, and quality of life; and (2) investigate the potential influence of moderating factors, including control types and intervention parameters such as session duration, frequency, and total duration.

## 2 Methods

### 2.1 Research design

The study strictly adhered to the Preferred Reporting Items for Systematic Reviews and Meta-Analyses (PRISMA) guidelines (27). The PRISMA checklist for reporting the meta-analysis results is shown in Supplementary Table 1. Additionally, the study protocol was pre-registered with the international prospective systematic evaluation registry PROSPERO (registration number: CRD420251053317).

### 2.2 Search strategy

We systematically searched seven databases, including English-language databases (PubMed, EMBASE, Cochrane Library, Web of Science) and Chinese databases (CNKI, VIP, WanFang), from their inception through May 1, 2025. The search was repeated on September 28, 2025, without any language restrictions. Key search terms combined two topics: TCEs delivered as digital health technologies and RCTs. The complete search strategy is provided in Supplementary Table 2.

### 2.3 Eligibility criteria

We developed inclusion criteria based on the Cochrane PICOS framework:

(P) Participants: Older adults aged 60 and above were eligible if they met the following criteria: (1) no major cognitive impairment, defined as a Clinical Dementia Rating–Sum of Boxes score less than 3 and a Montreal Cognitive Assessment score above 20; (2) no diagnosis of dementia, Parkinson’s disease, significant post-stroke cognitive impairment, cognitive impairment following traumatic brain injury, or other neurodegenerative disorders; and (3) no major mobility limitations that restrict walking or basic physical activities (e.g., fractures, osteoarthritis, joint replacements, amputations, or uncontrolled cardiovascular disease).

(I) Intervention: TCEs delivered through digital health technologies (e.g., AI, VR, telemedicine).

(C) Comparator: Inactive controls (treatment as usual) or active controls (sham or traditional face-to-face therapy).

(O) Outcome Measure: Included trials must report at least one of the following outcome categories: physical functioning, cognitive functioning, mental health, or quality of life.

(S) Study design: Only RCTs are considered.

Exclusion criteria included the following: (1) quasi-randomized studies, reviews, conference proceedings, patents, secondary analyses, and case reports; (2) hospitalized participants; and (3) TCEs that were conducted independently of digital health technologies.

### 2.4 Data extraction

Two independent reviewers screened the titles, abstracts, and full texts of the studies. Any discrepancies in their assessments were resolved through consensus meetings involving a third reviewer. The following information was independently extracted from eligible studies: year of publication, country, study design (including sample size, target population, control type, exercise intervention parameters, and outcomes), sample characteristics (age), and data necessary for calculating effect sizes (mean and standard deviation). If the standard deviation was not reported, it was calculated from the standard error, confidence interval, or interquartile range of the group mean, using the formula provided in the Cochrane Handbook (28).

### 2.5 Quality assessment

We assessed the methodological quality of the included RCTs using the Cochrane Risk of Bias tool (29). The following domains were evaluated: random sequence generation, allocation concealment, blinding of participants and personnel, blinding of outcome assessment, incomplete outcome data, selective reporting, and other bias. Each domain was rated as ‘low,’ ‘unclear,’ or ‘high’ risk of bias. The overall quality of evidence was assessed using the Grading of Recommendations, Assessment, Development, and Evaluation (GRADE) approach (30). RCTs were initially assigned a ‘high’ quality rating, which could be downgraded based on factors such as risk of bias, inconsistency, indirectness, imprecision, and publication bias. Both assessments were conducted independently by two reviewers, with any disagreements resolved through consensus involving a third reviewer.

### 2.6 Statistical analysis

Meta-analysis was conducted using RevMan 5.3 and Stata 17.0 software. The outcome data were continuous variables. When measurement tools and units were consistent, the weighted mean difference (WMD) was used; otherwise, the standardized mean difference (SMD) was applied and interpreted according to Hedges’ (adjusted) g criteria: small (0.2–0.5), medium (0.5–0.8), and large (>0.8) (31). For multi-arm trials involving multiple common intervention groups, the sample size was divided among the comparison groups to prevent inflation of statistical power (31). Heterogeneity was assessed using Cochran’s Q test and quantified with the I^2^ statistic; an I^2^ value 50% indicated substantial heterogeneity, necessitating the use of a random-effects model; otherwise, a fixed-effects model was applied. Pooled effects were visualized with forest plots, and sources of heterogeneity were explored through subgroup analyses. Publication bias was evaluated using funnel plots and Egger’s test (32). Sensitivity analysis was conducted to evaluate the influence of each included study on the pooled effect size using a leave-one-out approach.

### 2.7 Subgroup analysis

To explore sources of heterogeneity and moderators of efficacy, subgroup analyses were conducted based on control type and exercise intervention parameters. Control types were classified according to Sîrbu and David (33) as follows: inactive controls (including conventional treatment and health education that provide minimal control and are susceptible to placebo effects); nonspecific active controls (which resemble the trial group intervention but lack a core component, such as stretching exercises or fitness walking); and specific active controls (validated non-digitalized TCEs). Exercise dosage parameters were categorized as follows: single-session duration (short: ≤ 40 min; long: > 40 to ≤ 60 min), frequency (once, twice, or thrice per week), and total duration (short-term: < 24 weeks; long-term: ≥ 24 weeks).

## 3 Results

### 3.1 Search results

**Figure 1:**
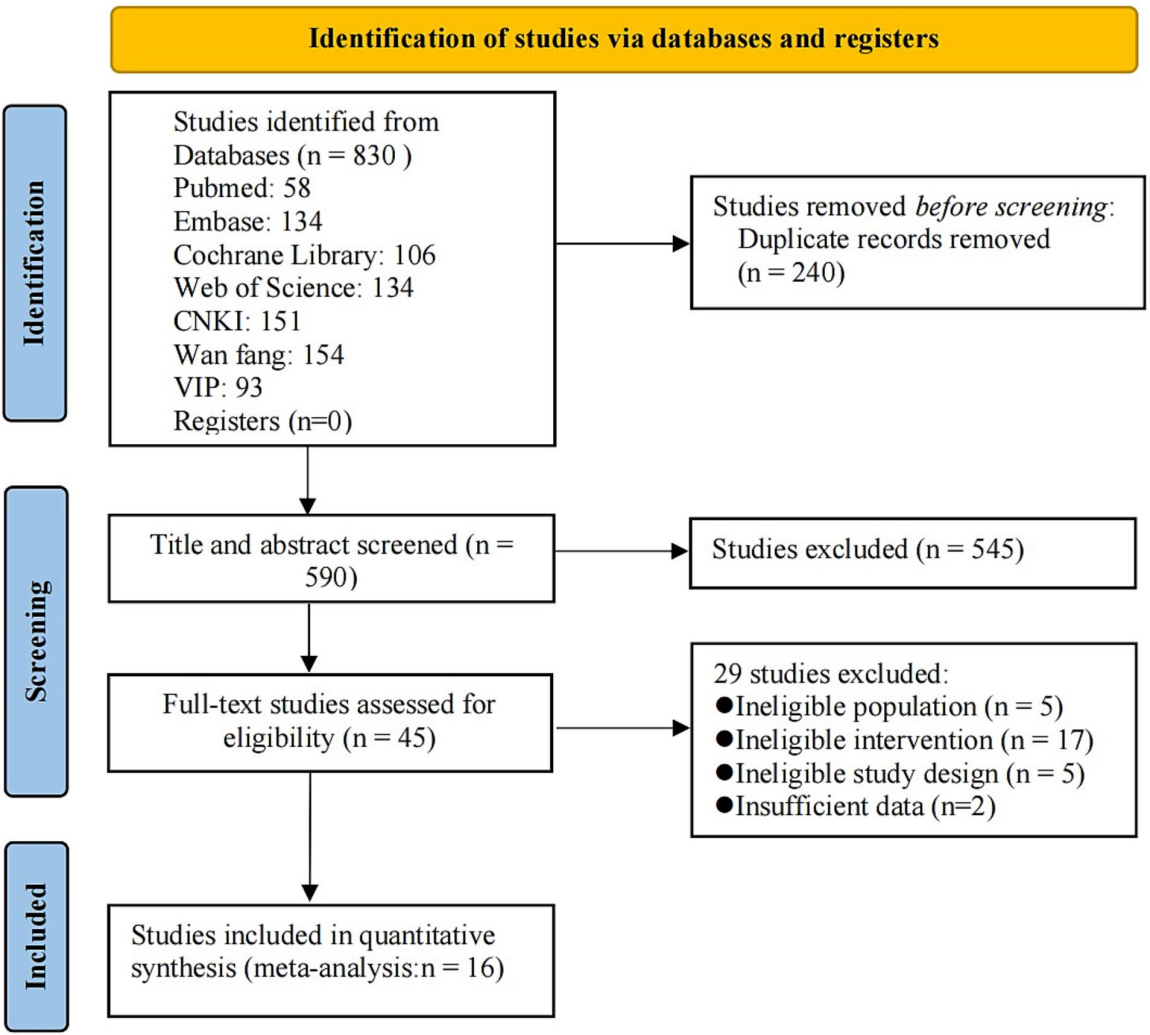
PRISMA flow diagram of study selection.

The initial search process yielded a total of 830 articles. After removing duplicate citations, 590 studies were screened based on their titles and abstracts, of which 45 were deemed potentially eligible for full-text review. Ultimately, 16 eligible trials (34–49) were selected for this systematic evaluation and meta-analysis (see Figure 1). The remaining 29 studies (see Supplementary Table 3) were excluded for the following reasons: ineligible population (*n* = 5), ineligible intervention (*n* = 17), ineligible study design (*n* = 5), and insufficient data (*n* = 2).

### 3.2 Characteristics of included studies

A total of 16 RCTs were included in this study; 13 were conducted in China, and 3 were conducted in the United States. The sample sizes ranged from 28 to 318 participants, with a cumulative total of 1,604 cases and a mean age range of 62 to 76 years. Among the various types of digital health delivery that enhanced TCEs, a total of 7 studies (43.8%) utilized remote videoconferencing, 5 studies (31.3%) employed AI-based movement correction applications, 6 studies (37.5%) utilized VR-enhanced technology, and 1 study (6.3%) utilized exergaming. The primary outcomes assessed included functional mobility, balance, grip strength, cognitive function, levels of depression, and quality of life. Detailed characteristics of the included studies are presented in Supplementary Table 4.

### 3.3 Quality assessment

**Figure 2:**
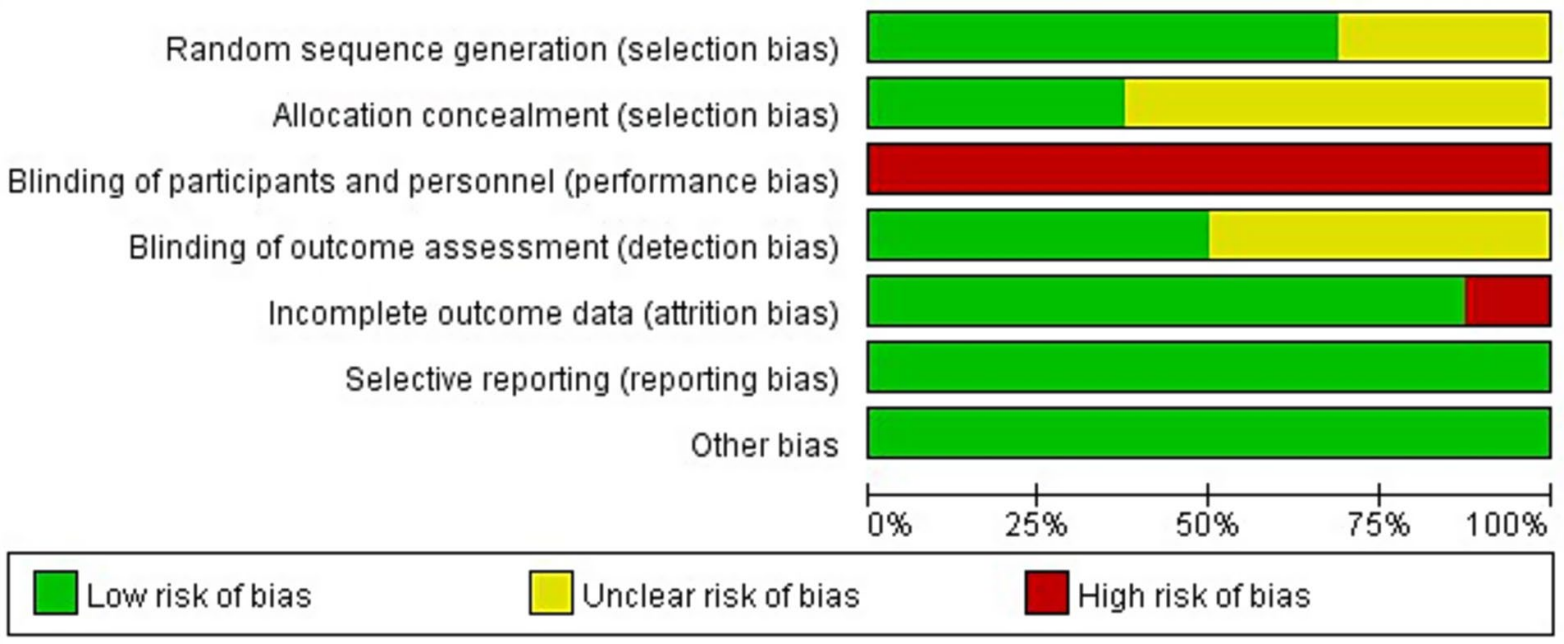
Graphical map of RCT bias analysis.

**Figure 3:**
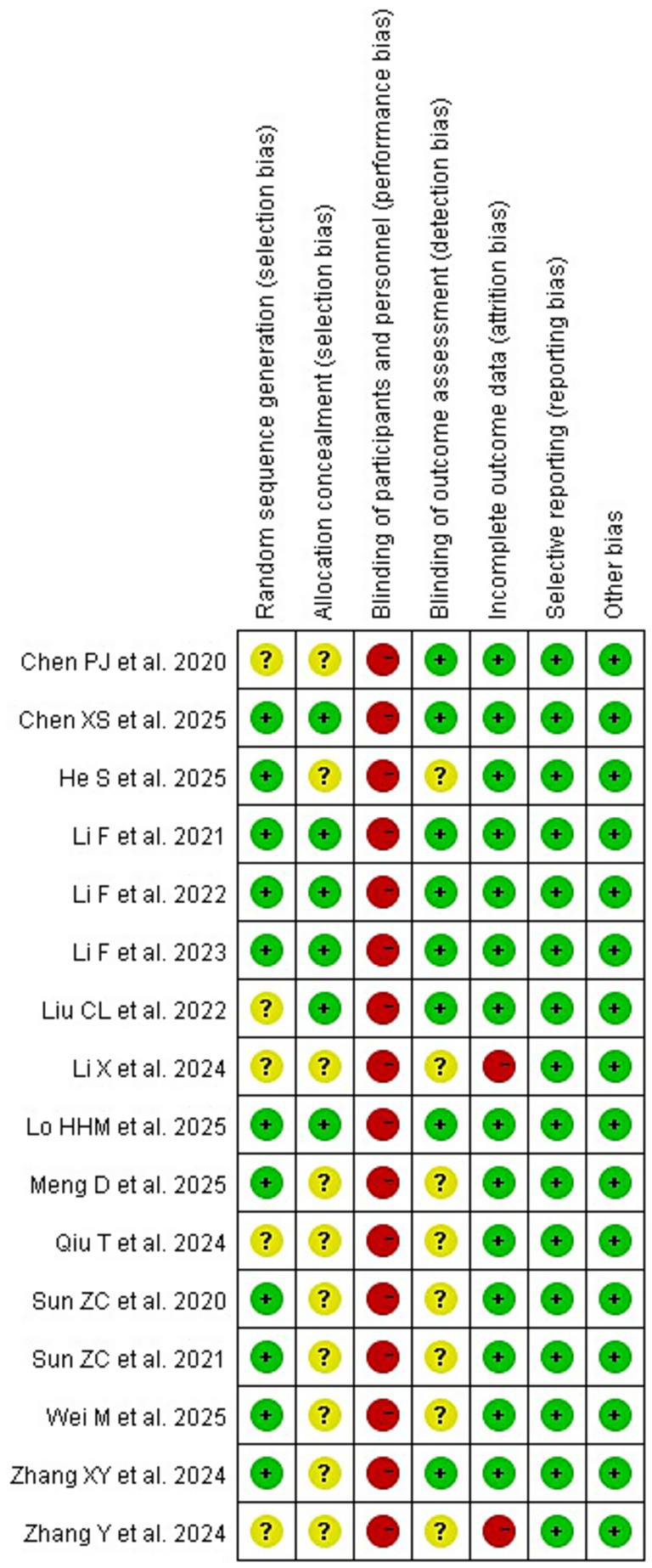
Summary map of RCT bias analysis.

For the 16 included studies, we provide a summary of the assessment results along with corresponding graphs (see Figures 2, 3). All studies were assessed as having a high risk of performance bias (100%) due to inherent limitations in the intervention, which prevented blinding of participants and personnel. Due to the lack of specific methods or missing information, 10 studies (62.5%) on allocation concealment, 8 studies (50%) on blinding of outcome assessment, and 5 studies (31.3%) on random sequence generation were all judged to have an unclear risk of bias. Other areas demonstrated a low risk of bias: incomplete outcome data (87.5%), selective reporting (100%), and other biases (100%). All outcomes were evaluated using the GRADE system, which indicated a certainty of evidence ranging from very low to moderate (see Supplementary Table 5).

### 3.4 Outcome statistics

The measurements were visualized using forest plots, and subgroup analyses were conducted. Specific results of the subgroup analyses are presented in Supplementary Table 6. The forest plots of the pooled results are presented in Supplementary Figure 1, while the plots for the subgroup analyses are shown in Supplementary Figures 2–5.

### 3.5 Physical function

#### 3.5.1 Functional mobility

A random-effects meta-analysis involving ten studies and 600 participants found that digitalized TCEs significantly improved functional mobility (WMD = −0.81; 95% CI, −1.28 to −0.33; *p* = 0.0009; Supplementary Figure 1). However, there was a high degree of heterogeneity (I^2^ = 76%), and the certainty of the evidence was low.

Subgroup analyses indicated that the parameters of exercise interventions and the type of control group served as significant moderators. Specifically, the effect size was greatest when the duration of a single session exceeded 40 min, the frequency was twice per week, and the total duration was 24 weeks or more (WMD = −1.62; 95% CI, −2.14 to −1.11; *p* < 0.00001; I^2^ = 51%; Supplementary Table 6). Additionally, a larger effect was observed in the nonspecific active control group (WMD = −1.62; 95% CI, −2.14 to −1.11; *p* < 0.00001; I^2^ = 51%; Supplementary Table 6) compared to the specific active control group.

#### 3.5.2 Balance function

A fixed-effects meta-analysis involving seven studies and 468 participants found that digitalized TCEs significantly improved balance function (SMD = 0.88; 95% CI, 0.68 to 1.08; p < 0.00001; Supplementary Figure 1), with negligible heterogeneity (I^2^ = 0%). The certainty of the evidence was moderate. Subgroup analyses indicated that neither the type of control nor the single session, frequency, and duration of the intervention were significantly associated with the effect size on balance function (Supplementary Table 6).

#### 3.5.3 Grip strength

A fixed-effects meta-analysis involving nine studies and 316 participants revealed that digitalized TCEs did not significantly enhance grip strength (WMD = −0.26; 95% CI, −1.15 to 0.62; *p* = 0.56; Supplementary Figure 1), and no heterogeneity was observed (I^2^ = 0%). The certainty of the evidence is low.

### 3.6 Cognitive function

A random-effects meta-analysis involving eleven studies and 801 participants demonstrated that digitalized TCEs significantly enhanced global cognitive function (WMD = 1.98; 95% CI, 1.44 to 2.52; *p* < 0.00001; Supplementary Figure 1). However, the analysis revealed high heterogeneity (I^2^ = 84%), and the certainty of the evidence was rated as low.

Subgroup analyses revealed that differences in effect size were associated with the type of control and the duration of the intervention, but not with session length or frequency. Compared with the nonspecific and specific active control groups, a larger effect size was observed in the inactive control group (WMD = 2.70; 95% CI, 1.26 to 4.15; *p* = 0.0002; I^2^ = 87%; Supplementary Table 6). Additionally, the intervention effect size lasting 24 weeks or more (WMD = 2.70; 95% CI, 1.70 to 3.71; *p* < 0.00001; I^2^ = 87%; Supplementary Table 6) was significantly greater than that observed within 24 weeks.

### 3.7 Depression

A random-effects meta-analysis involving eight studies and 779 participants found that digitalized TCEs did not significantly reduced depression levels (SMD = −0.71; 95% CI, −1.48 to 0.05; *p* = 0.07; Supplementary Figure 1). The analysis revealed a high degree of heterogeneity (I^2^ = 95%), and the certainty of the evidence was rated as very low.

Subgroup analyses revealed that the type of control and the duration of the intervention—rather than the frequency—significantly influenced the effect sizes. Specifically, the effect size observed in the comparison with the inactive control (SMD = −3.83; 95% CI, −4.38 to −3.27; *p* < 0.00001; Supplementary Table 6) was significantly greater than that observed with the nonspecific active control (SMD = −0.53; 95% CI, −0.77 to −0.29; *p* < 0.0001; I^2^ = 0%; Supplementary Table 6) and the specific active control (SMD = −0.19; 95% CI, −0.67 to 0.29; *p* = 0.44; I^2^ = 70%; Supplementary Table 6). Furthermore, interventions lasting 24 weeks or longer produced a significantly greater effect (SMD = −1.39; 95% CI, −2.57 to −0.21; *p* = 0.02; I^2^ = 97%; Supplementary Table 6) compared to shorter interventions.

### 3.8 Quality of life

A fixed-effects meta-analysis involving sixteen studies and 982 participants demonstrated that digitalized TCEs only marginally improved the quality of life (SMD = 0.17; 95% CI, 0.03 to 0.30; *p* = 0.01; Supplementary Figure 1). The analysis revealed low heterogeneity (I^2^ = 3%) and provided evidence of moderate certainty.

Subgroup analyses revealed that the type of control was significantly associated with differences in effect sizes, whereas the duration of a single session, frequency, and total intervention duration showed no significant associations. Specifically, the inactive control group exhibited a larger effect size (SMD = 0.60; 95% CI, 0.31 to 0.89; *p* < 0.0001; I^2^ = 9%; Supplementary Table 6) compared to both nonspecific and specific active control groups.

### 3.9 Sensitivity analysis and publication bias

As shown in Supplementary Figure 6, we performed a leave-one-out analysis to examine the robustness of the results. The results indicate that the combined effect sizes and their 95% confidence intervals for functional mobility, balance, grip strength, and cognitive function remained largely unchanged after each study was sequentially excluded. The direction of the effects remained consistent, and the statistical significance was maintained. However, for the depression outcome, removing the study by Qiu et al. (44) reduced the I^2^ value to 61%, and altered the statistical significance (*p* = 0.02). Similarly, for the quality of life outcome, excluding the study by Sun et al. (45) lowered the I^2^ value to 0%, and changed the statistical significance (*p* = 0.09). These two studies (44, 45) critically influenced the robustness of their respective results.

Funnel plots and Egger’s test revealed no evidence of publication bias in the results presented above (Supplementary Figure 7).

### 3.10 Adverse events and follow-up reports

None of the 16 studies included in this review reported moderate or serious adverse events associated with the intervention, indicating a favorable overall safety profile. Specifically, four studies (35, 37, 38, 40) reported several mild adverse events, including hernia pain, musculoskeletal discomfort, fatigue, and eyestrain. The remaining 12 studies reported no adverse events. Only one study (37) conducted a follow-up assessment after the 24-week intervention, demonstrating that the effects on cognitive function, functional mobility, and balance were sustained for up to 48 weeks.

## 4 Discussion

### 4.1 Principal findings

This study integrates digital health technologies with TCEs, proposing a novel intervention model termed “digitalized TCEs.” Through a systematic review and meta-analysis, it evaluates their role in promoting healthy aging among older adults. Results from 16 included studies indicate that digitalized TCEs have positive effects on improving functional mobility, balance, cognition, and quality of life (*p* < 0.05). However, no significant benefits were observed for depressive symptoms or grip strength (*p* > 0.05). Given that the overall certainty of the evidence ranges from very low to moderate, these conclusions should be considered preliminary and require validation through larger-scale, high-quality studies.

Specifically, digitalized TCEs provide low to moderate certainty evidence supporting significant improvements in functional mobility (WMD = −0.81), balance (SMD = 0.88), and cognition (WMD = 1.98). These effects are likely attributable to the mind–body coordination mechanisms inherent in TCEs and the cognitive enhancement facilitated by digital technology. TCEs require participants to perform complex movement sequences, slow movements in a half-squat posture, and cognitive tasks (e.g., action memory, visuospatial perception coordination), thereby simultaneously activating motor and cognition-related brain regions (15). This effectively enhances functional mobility, balance (13, 50), and cognitive function in older adults (51). Additionally, digital technology further promotes the mobilization of cognitive resources—including attention, memory, and decision-making abilities—through dynamic virtual environments and evolving task demands, thereby facilitating sensorimotor integration (52).

Notably, based on low-certainty evidence, digitalized TCEs did not produce significant improvements in grip strength. This outcome may be explained by three factors. First, TCEs primarily involve low-intensity, whole-body coordination training (e.g., breath-guided and slow stretching movements), indicating that their training model may lack the high-intensity resistance necessary to stimulate the upper limb muscles responsible for grasping (53, 54). Second, while digital augmentation technologies enhance psychophysical benefits through audiovisual feedback and immersive environments, they provide insufficient physiological stimulation for fine motor skills of the upper limbs (55). Third, the average intervention duration of 10 weeks across the included RCTs may be too short to elicit significant muscle strength adaptations.

Regarding depressive symptoms, this study did not observe a significant improvement; however, the certainty of the evidence was very low, and sensitivity analyses indicated that this result was unstable. This finding contrasts with previous meta-analyses that confirmed the antidepressant effects of TCEs (14) and with reports suggesting that digital technologies enhance psychological benefits by creating immersive environments and providing real-time guided feedback (56, 57). This inconsistency may be attributed to several factors. First, TCEs-mediated improvements in depression involve enhanced functional connectivity in specific brain regions and endocrine regulatory mechanisms (58, 59). Such effects on brain plasticity and endocrine homeostasis—both critical for emotional stability—require prolonged intervention to manifest (14). The average duration of the RCTs included in this study was 16 weeks, which may have limited the cumulative emotional benefits. Second, digital remote exercise may lack interpersonal interaction and emotional engagement (60), potentially diminishing its impact on emotional regulation. Similarly, although quality of life showed a statistically significant improvement, the effect size was small (SMD = 0.17). Quality of life encompasses multiple dimensions, including physical health, psychological well-being, and social participation. Exercise interventions may require long-term implementation to fully exert their effects (61). The small effect size observed may reflect the limited psychological benefits of the current intervention, as the relatively short duration was insufficient to comprehensively influence all domains. Furthermore, the absence of social participation in digital remote exercise (60) likely contributed to the overall weaker effect.

In summary, this study suggests that digitalized TCEs represent a promising strategy for healthy aging, particularly in enhancing physical and cognitive functions. Future research should extend intervention durations (e.g., ≥24 weeks) and improve the design of remote interpersonal interactions to validate the long-term benefits for emotional well-being and quality of life. Furthermore, the observed heterogeneity may result from differences in control types and exercise parameters, warranting further investigation through subsequent subgroup analyses.

### 4.2 Subgroup analysis by control type

Subgroup analyses indicated that the type of control significantly influenced the assessment of efficacy. In terms of improving functional mobility, digitalized TCEs demonstrated a significantly larger effect size in comparison with nonspecific active controls, such as sham therapy (WMD = −1.62). However, when compared with specific active controls, such as non-digitalized TCEs, the effect size decreased (WMD = −0.40). Similar trends were observed for cognitive function, depression, and quality of life indicators. Compared with inactive controls, such as treatment as usual, digitalized TCEs showed significantly larger effect sizes (cognitive function, WMD = 2.70; depression, SMD = −3.83; quality of life, SMD = 0.60). When compared with nonspecific active controls, the effect sizes were smaller (cognitive function, WMD = 1.77; depression, SMD = −0.53; quality of life, SMD = 0.10). Compared to specific activity controls, the effect sizes further decreased (cognitive function, WMD = 1.14; depression, SMD = −0.19; quality of life, SMD = 0.01). This finding aligns with the conclusions drawn in the study by Sîrbu and David (33), which emphasizes the importance of considering the activity level of the control type when evaluating efficacy. The gradient of effect sizes suggests that digitalized TCEs remain non-inferior when the control condition includes active therapeutic components (e.g., TCEs, stretching exercises, and fitness walking), and that the efficacy advantage is more pronounced in inactive controls (e.g., treatment as usual) that exclude both specific and nonspecific factors.

### 4.3 Subgroup analysis of dosage effects

Subgroup analyses revealed dose–response relationships for exercise parameters, including single-session duration, frequency, and total duration. The results indicate that long-term interventions (≥24 weeks) produced the greatest effect sizes across all domains, including functional mobility (WMD = −1.62), cognitive function (WMD = 2.70), depression (SMD = −1.39), and quality of life (SMD = 0.30). Furthermore, exercise sessions lasting more than 40 min demonstrated significant improvements in functional mobility (WMD = −1.62), cognitive function (WMD = 2.12), and quality of life (SMD = 0.25). In contrast, short-term interventions (<24 weeks) generally yielded smaller effect sizes, particularly for functional mobility (WMD = −0.40), cognitive function (WMD = 1.41), depression (SMD = 0.02), and quality of life (SMD = 0.06). Interventions with single-session durations of 40 min or less also showed diminished effects, with significantly reduced effect sizes across relevant domains (functional mobility: WMD = −0.40; cognitive function: WMD = 0.88; quality of life: SMD = 0.08). This dose-dependent difference may result from the dual effects of long-term exercise accumulation: on one hand, exercise enhances neuromuscular coordination efficiency through a neuromuscular adaptive remodeling process that requires prolonged accumulation (62, 63); on the other hand, the regulation of brain plasticity and endocrine homeostasis, which influence emotional stability, also necessitates long-term exercise accumulation to be effective (14).

Additionally, subgroup analyses revealed that the effect size on balance function was not influenced by exercise dosage parameters, including single-session duration, frequency, or intervention duration. This finding may be attributed to the rapid improvement in balance function, which plateaus after short-term interventions. A study by Li et al. (13) supports this observation: TCEs rapidly improve balance function within short-term interventions (≤12 weeks), whereas long-term interventions (≥20 weeks) primarily serve to maintain rather than further enhance these gains.

In subgroup analyses examining intervention frequency, exercise frequency did not significantly moderate health outcomes, except for functional mobility. Interestingly, exercising thrice per week (WMD = −0.40) was less effective than exercising twice per week (WMD = −1.62) in enhancing functional mobility among older adults.

In summary, both the duration of the intervention and the length of individual sessions jointly influence the effectiveness of exercise programs. Long-term interventions with sufficiently extended sessions may be crucial for improving multidimensional health outcomes in older adults. Notably, digital interventions face challenges in maintaining participants’ long-term motivation and adherence (64). Therefore, future research should not only rigorously assess participant compliance but also actively explore strategies to enhance sustained engagement in digitalized TCEs.

### 4.4 Analysis of sources of heterogeneity

Meta-analysis reveals significant heterogeneity in intervention outcomes for functional mobility, cognition, and depression. Subgroup analyses suggest that improvements in these measures may depend on cumulative effects from long-term exercise, with both the overall duration of the intervention and the length of individual sessions significantly influencing effect sizes. These factors likely contribute substantially to the observed heterogeneity. Additionally, the type of control group (e.g., inactive, nonspecific active, or specific active) moderates the results, further increasing variability across studies.

Sensitivity analysis revealed that intervention duration and control type may be key factors influencing effect sizes. For example, in the study by Qiu et al. (44), the intervention effect size was largest in the inactive control group, significantly affecting the heterogeneity and direction of depression outcomes. Similarly, the study by Sun et al. (45) reported the largest effect size, attributed to the longest intervention duration, which also influenced the direction of quality of life outcomes.

Moreover, the digital technology proficiency of older participants and the level of guidance and support provided by various digital intervention models may also influence intervention outcomes. Future studies should further investigate the mechanisms through which these variables affect effect sizes to establish a theoretical foundation for optimizing intervention strategies and reducing heterogeneity.

### 4.5 Practical implications

The findings of this study are of low certainty and should be considered preliminary. However, they provide valuable guidance for future research and clinical practice. The results support the feasibility of digitalized TCEs as an intervention for healthy aging. The application scenarios primarily encompass three aspects. First, in community settings such as senior activity centers, VR, remote video guidance, or AI technologies can be employed to offer diverse forms of group exercise, significantly enhancing participation, adherence, and user experience. Second, as a home-based rehabilitation program, digitalized TCEs can provide standardized rehabilitation guidance for older adults with stable conditions post-discharge, addressing the accessibility limitations of traditional rehabilitation services. Finally, within the context of home-based aging—particularly for older adults with limited transportation options or those living in remote areas—digitalized TCEs can overcome geographical barriers by establishing a sustainable platform for physical and mental exercises, thereby promoting the long-term maintenance of healthy behaviors.

However, several studies have identified obstacles in the implementation process, including the cost of hardware, the need for stable internet access, difficulties experienced by older adults in operating digital devices, and the requirement for technical support (65–67). To enhance the feasibility and effectiveness of the intervention, it is recommended to establish supportive frameworks, such as providing technology training for older adults, implementing equipment lending programs, and developing user-friendly interfaces specifically tailored to this population.

### 4.6 Limitations

This study has several limitations that should be considered when interpreting the results and planning future research. First, regarding methodological quality, none of the included studies successfully blinded participants and personnel. Since the primary outcomes were mostly assessed subjectively, awareness of group allocation may have introduced bias. Second, although this meta-analysis included 16 studies, some subgroup analyses involved only a small number of studies, limiting the robustness of the findings and necessitating cautious interpretation. Third, the literature included in this study was sourced exclusively from China and the United States, lacking evidence from other regions and ethnic groups. Therefore, the generalizability of the study’s conclusions remains uncertain. Finally, long-term follow-up data are relatively scarce in the existing studies, and the long-term effectiveness and safety of digitalized TCEs over extended periods require further validation.

Based on the limitations outlined above, future research should focus on improving blinding methods to enhance methodological rigor, conducting more long-term RCTs, and strengthening follow-up procedures to systematically evaluate the long-term benefits and sustainability of interventions. Additionally, expanding the geographical scope and increasing the diversity of study populations will improve the generalizability and practical applicability of the findings.

## 5 Conclusion

Amid the accelerating global aging trend, the WHO’s concept of healthy aging emphasizes maintaining functional capacity in older adults. Traditional health management models face challenges due to fragmented resources and insufficient continuity of care, creating an urgent need for innovation and transformation through digital health technologies. This systematic review and meta-analysis demonstrate that digitalized TCEs significantly improve older adults’ physical function, cognitive function, and quality of life. These findings align with the WHO’s health philosophy and offer promising intervention strategies for promoting healthy aging. Sustaining these health benefits requires ongoing participation. However, current evidence is limited by a lack of long-term efficacy data and low certainty, which restricts widespread adoption. Future research should focus on high-quality, long-term RCTs to explore the underlying mechanisms of these health effects and to refine safety assessment systems.

## Data Availability

The original contributions presented in the study are included in the article/Supplementary material, further inquiries can be directed to the corresponding author.
