# Supplementary material for: Effects of digitalized traditional Chinese exercises on the physical and mental health and quality of life of older adults: a systematic review and meta-analysis of randomized controlled trials
Source: Front Public Health. 2025 Dec 11;13:1725847. doi: 10.3389/fpubh.2025.1725847 (PMC12738345; doi:10.3389/fpubh.2025.1725847)
Supplement: Supplementary file 1 [file Table_1.docx]

Supplementary Material

Supplementary Figure 1. Forest Plot of the outcomes analysis 2

Supplementary Figure 2. Subgroup analysis by control type 4

Supplementary Figure 3. Subgroup analysis by duration of intervention 7

Supplementary Figure 4. Subgroup analysis by session duration 10

Supplementary Figure 5. Subgroup analysis by frequency of intervention 12

Supplementary Figure 6. Sensitive analysis 15

Supplementary Figure 7. Publication bias 19

Supplementary Table 1. PRISMA checklist. 25

Supplementary Table 2. Search strategies 28

Supplementary Table 3. Studies excluded by checking the full-text articles. 32

Supplementary Table 4. Characteristics of the included studies. 34

Supplementary Table 5. Certainty of evidence using GRADE. 37

Supplementary Table 6. Results of the analysis of individual outcome indicators and their subgroups. 38

#

# Supplementary Figure 1. Forest Plot of the outcomes analysis

Figure 1a: Functional Mobility


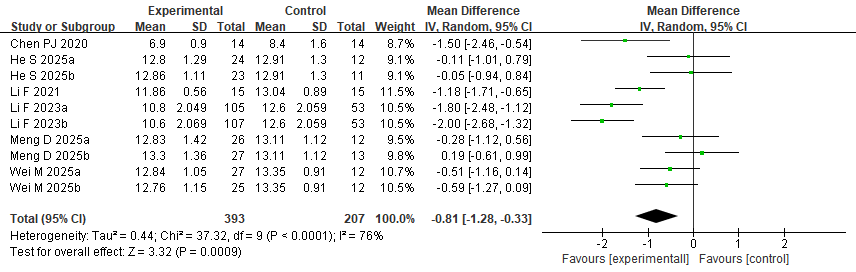


Figure 1b: Balance Function


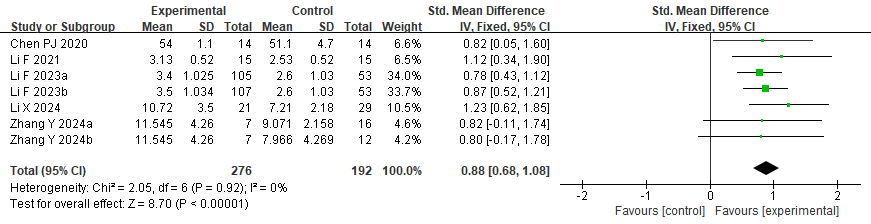


Figure 1c: Grip Strength


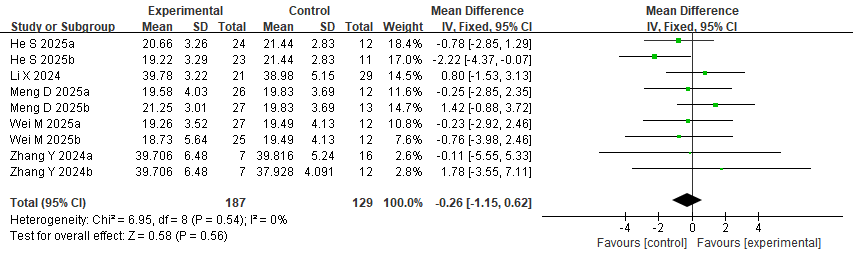


Figure 1d: Cognitive Function


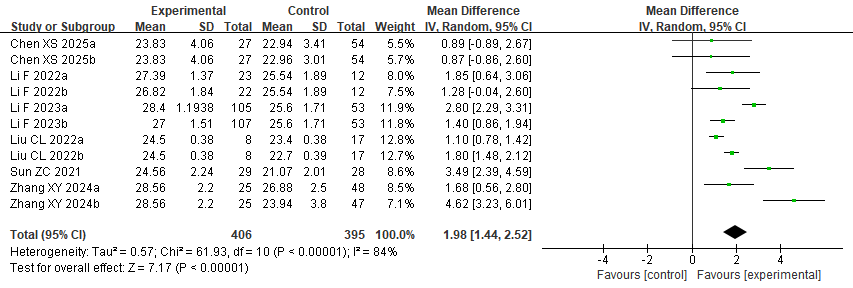


Figure 1e: Depression


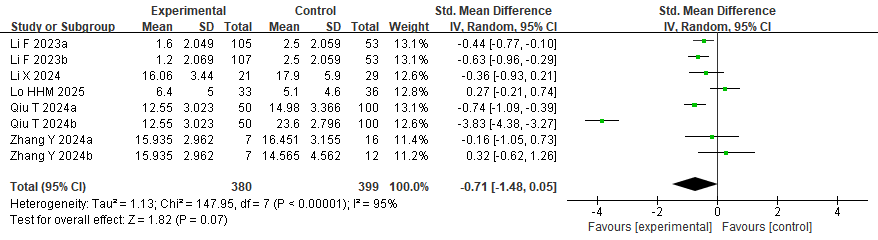


Figure 1f: Quality of Life


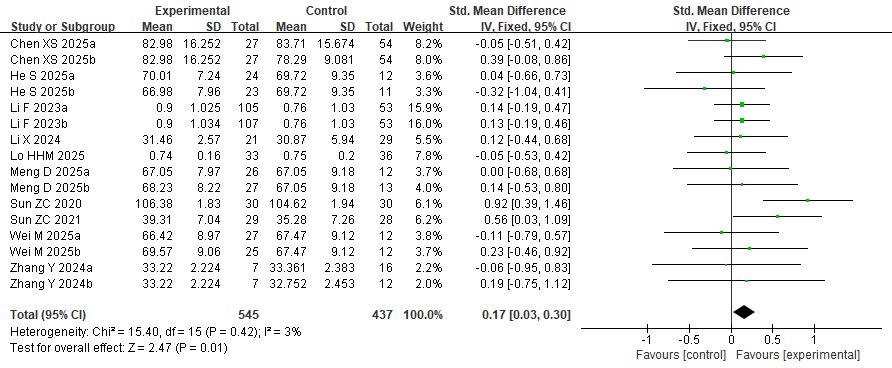


# Supplementary Figure 2. Subgroup analysis by control type

Figure 2a: Functional Mobility


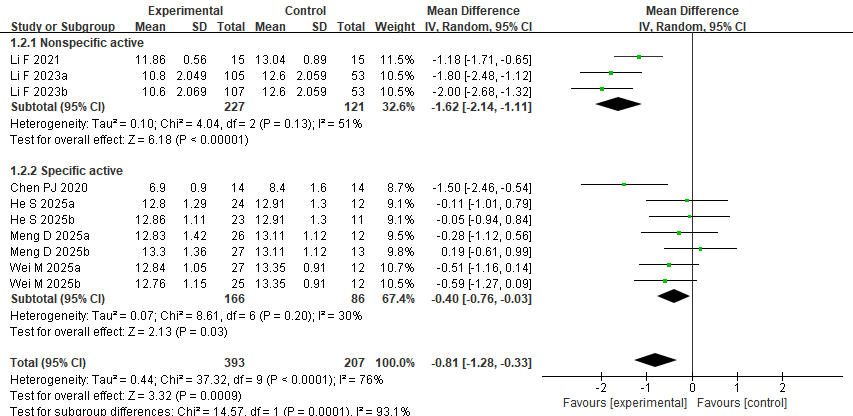


Figure 2b: Balance Function


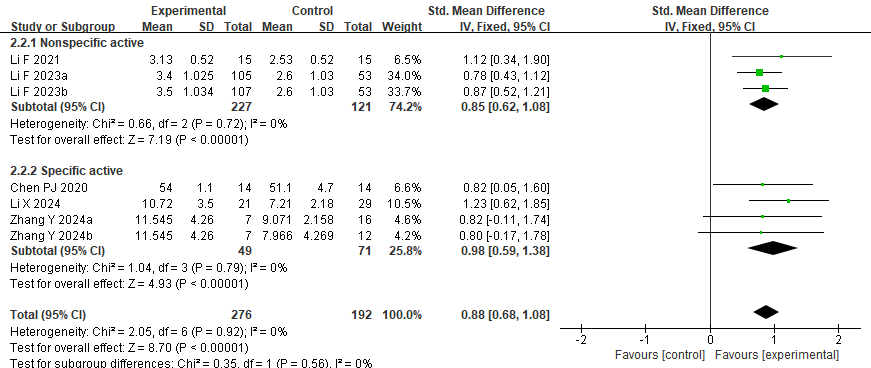


Figure 2c: Cognitive Function


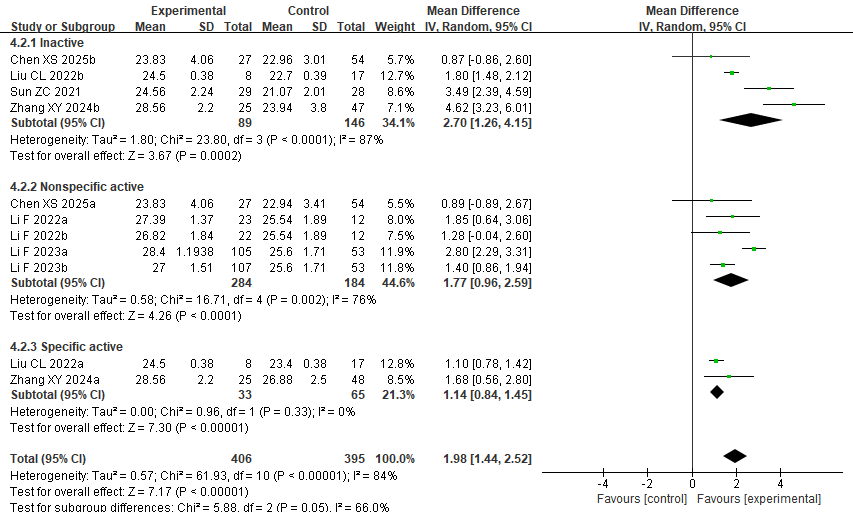


Figure 2d: Depression


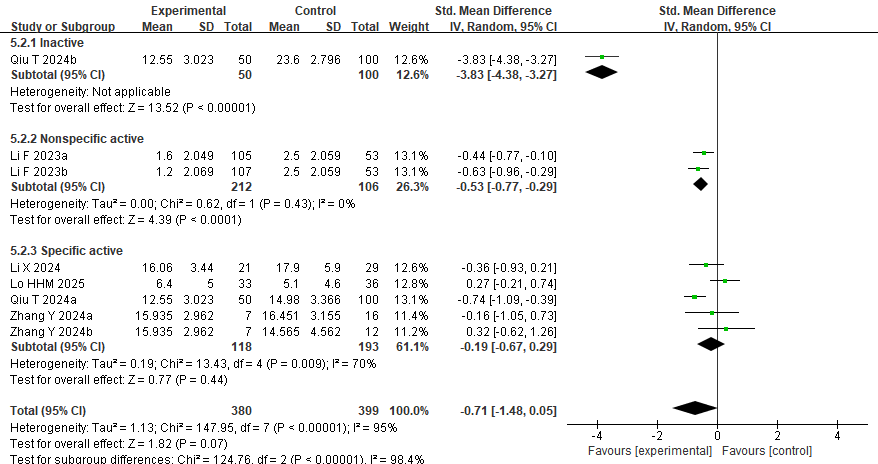


Figure 2e: Quality of Life


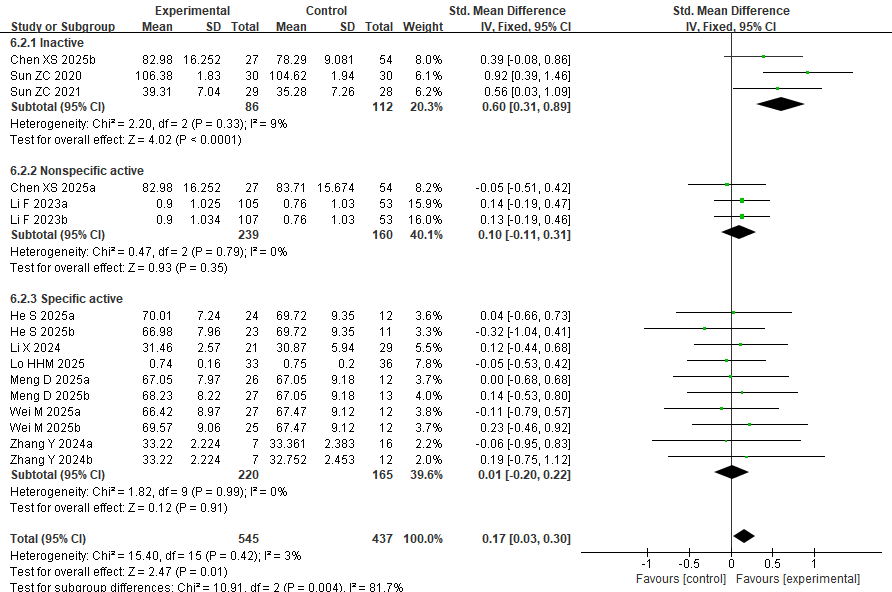


# Supplementary Figure 3. Subgroup analysis by duration of intervention

Figure 3a: Functional Mobility


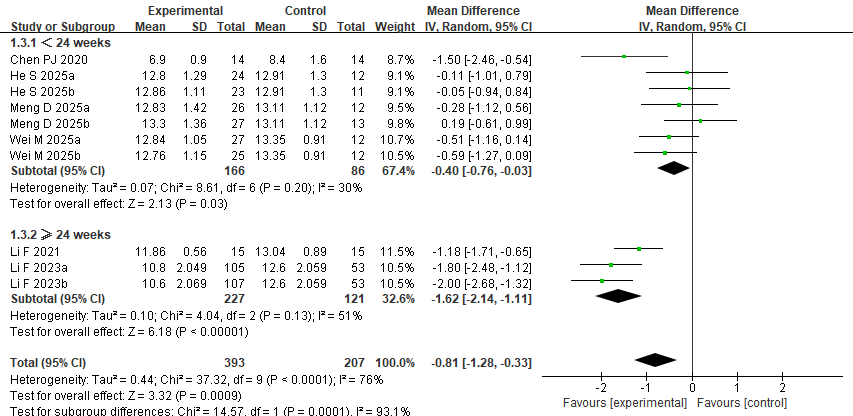


Figure 3b: Balance Function


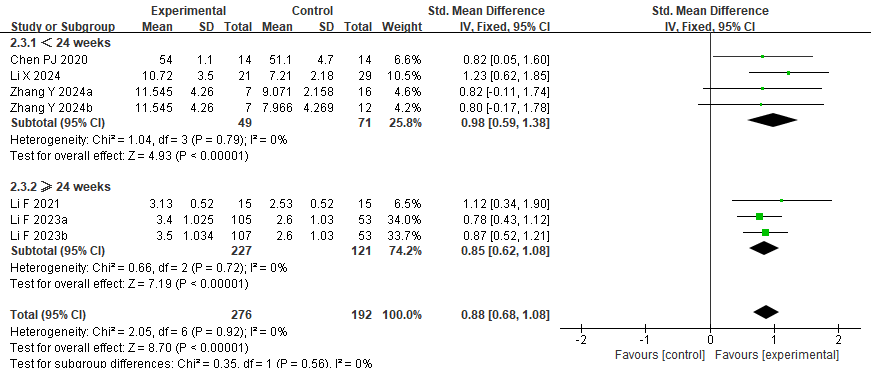


Figure 3c: Cognitive Function


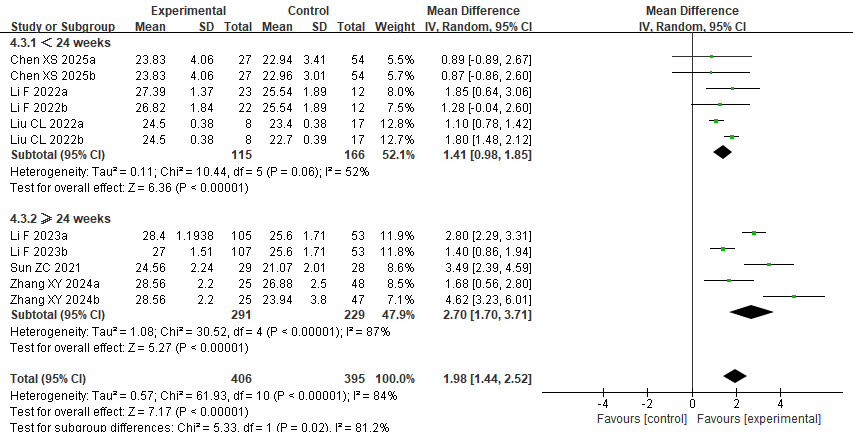


Figure 3d: Depression


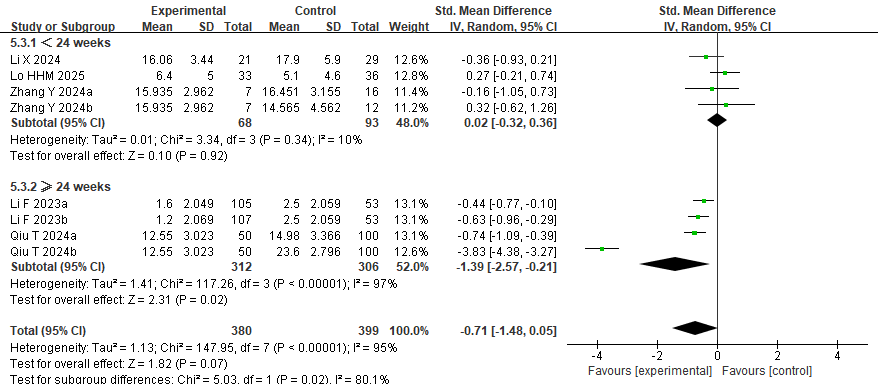


Figure 3e: Quality of Life


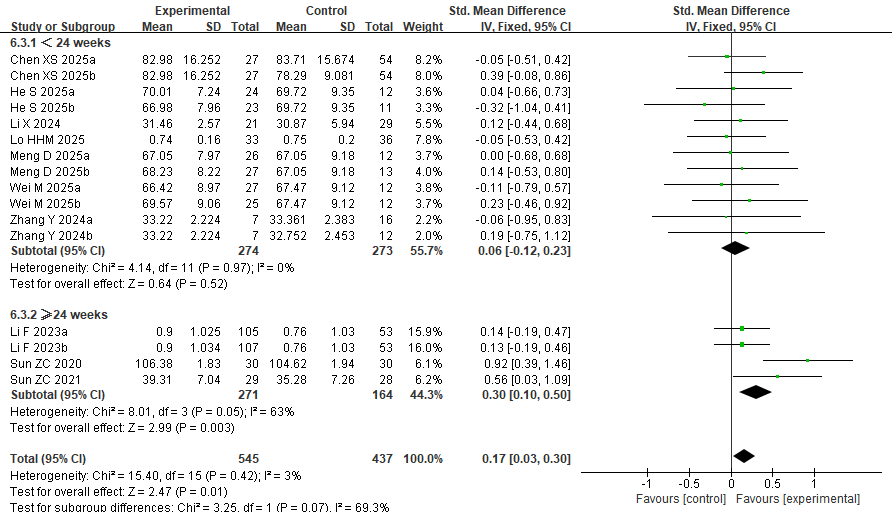


# Supplementary Figure 4. Subgroup analysis by session duration

Figure 4a: Functional Mobility


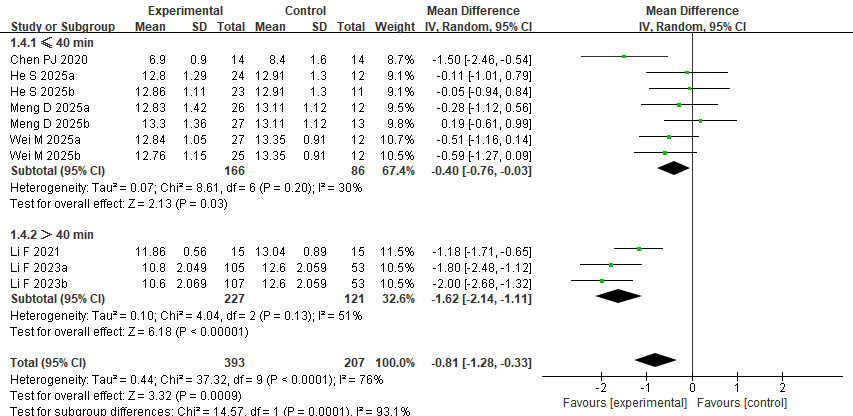


Figure 4b: Balance Function


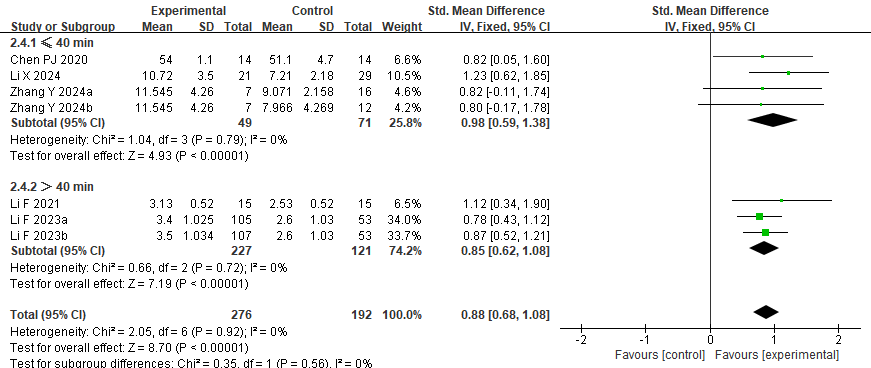


Figure 4c: Cognitive Function


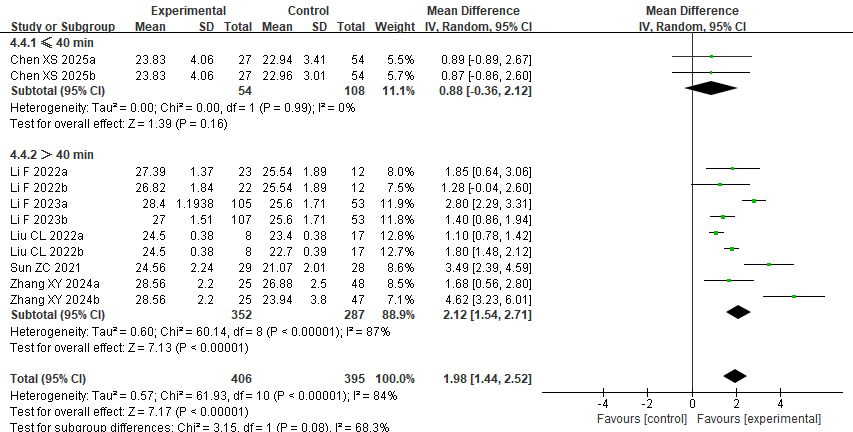


Figure 4d: Quality of Life


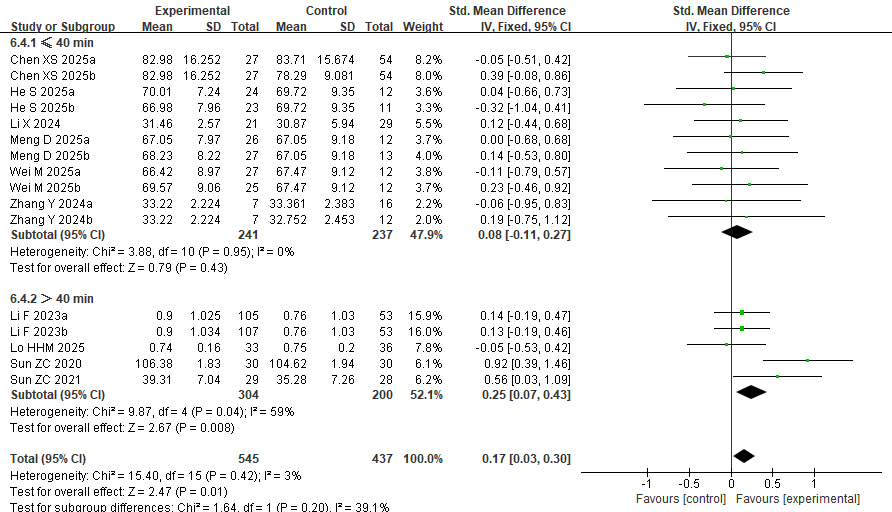


# Supplementary Figure 5. Subgroup analysis by frequency of intervention

Figure 5a: Functional Mobility


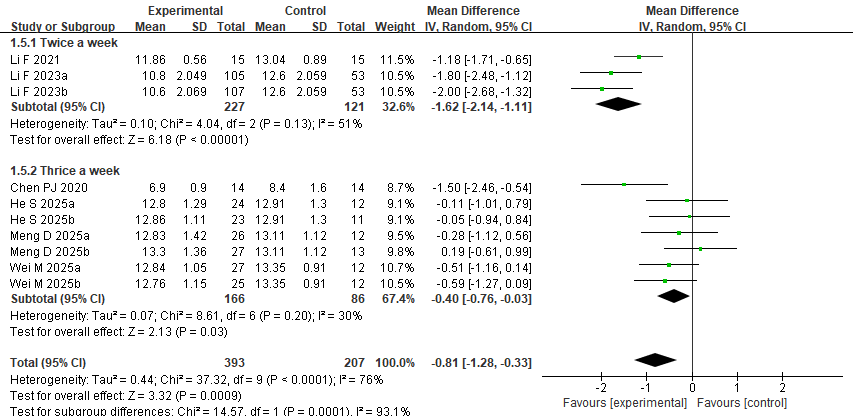


Figure 5b: Balance Function


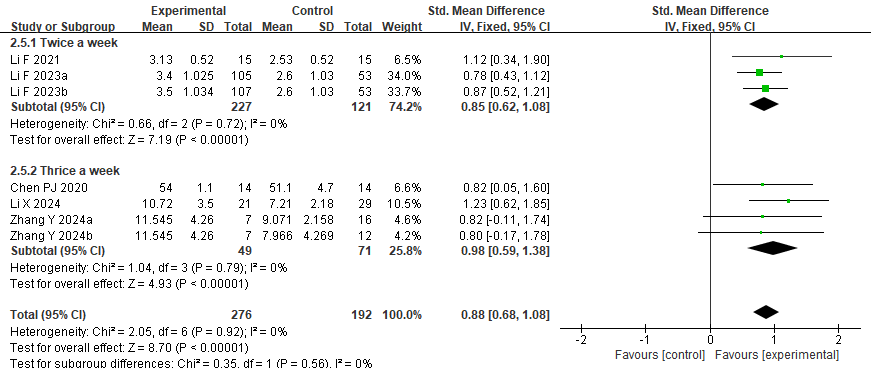


Figure 5c: Cognitive Function


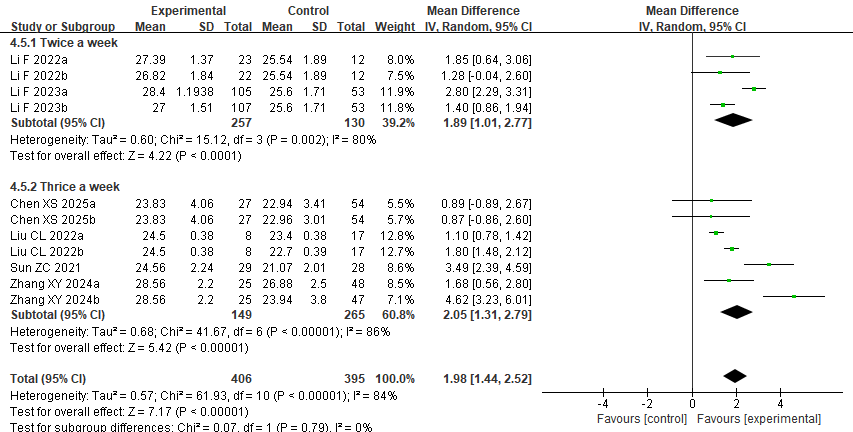


Figure 5d: Depression


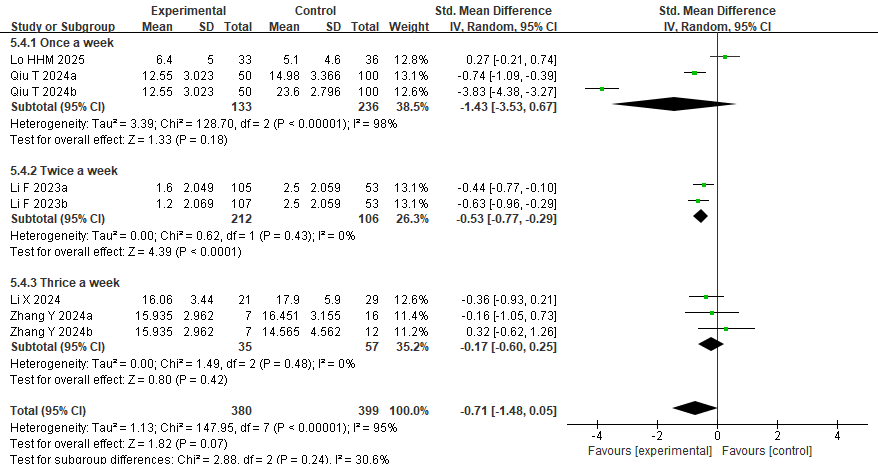


Figure 5e: Quality of Life


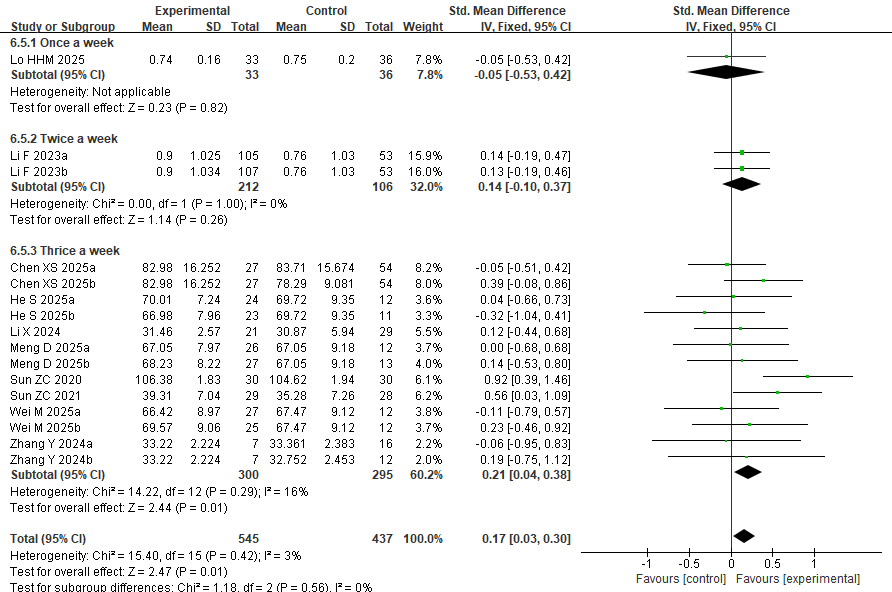


# Supplementary Figure 6. Sensitive analysis

Figure 6a: Functional Mobility

Figure 6b: Balance Function

Figure 6c: Grip Strength

Figure 6d: Cognitive Function

Figure 6e: Depression

Figure 6f: Quality of Life

# Supplementary Figure 7. Publication bias

Figure 7a: Funnel plots for functional mobility. This plot assesses publication bias in the functional mobility outcome. Egger's test indicates no evidence of publication bias for the functional mobility outcome (t = 1.26, p = 0.242).


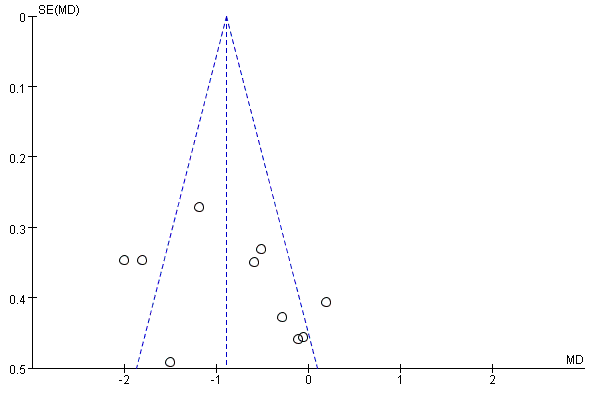


Figure 7b: Funnel plots for balance function. This plot assesses publication bias in the balance function outcome. Egger's test indicates no evidence of publication bias for the balance function outcome (t = 0.86, p = 0.427).


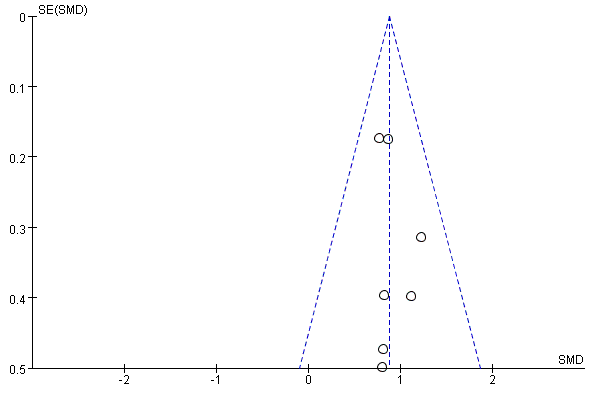


Figure 7c: Funnel plots for grip strength. This plot assesses publication bias in the grip strength outcome. Egger's test indicates no evidence of publication bias for the grip strength outcome (t = 0.71, p = 0.503).


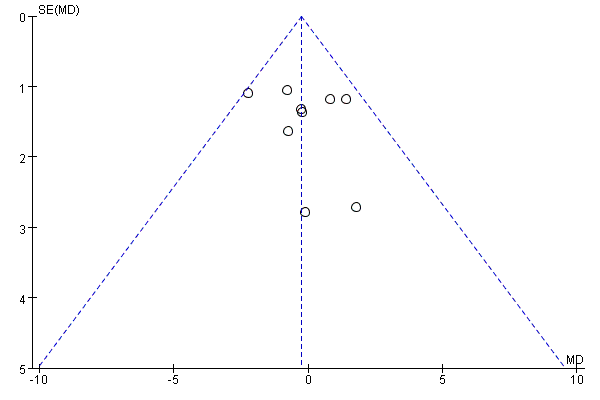


Figure 7d: Funnel plots for cognitive function. This plot assesses publication bias in the cognitive function outcome. Egger's test indicates no evidence of publication bias for the cognitive function outcome (t = 1.00, p = 0.344).


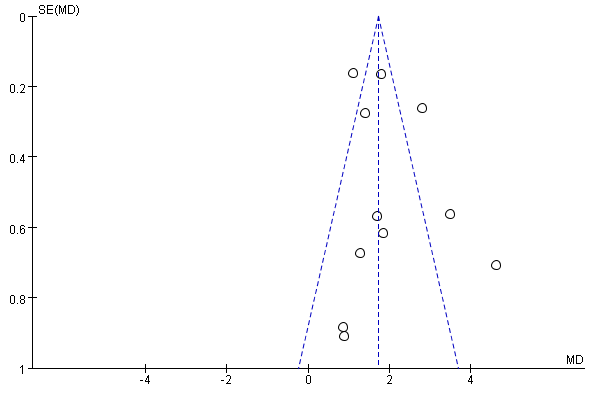


Figure 7e: Funnel plots for depression. This plot assesses publication bias in the depression outcome. Egger's test indicates no evidence of publication bias for the depression outcome (t = -0.12, p = 0.907).


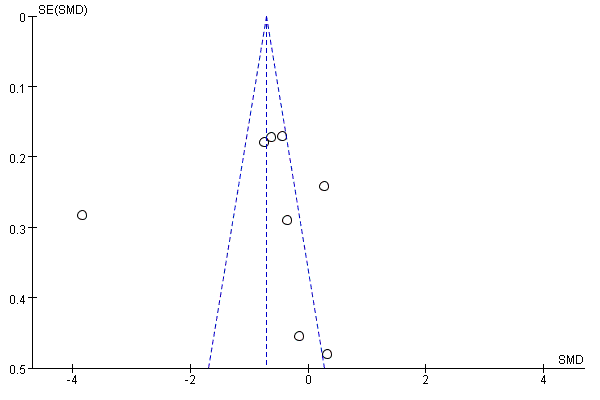


Figure 7f: Funnel plots for quality of life. This plot assesses publication bias in the quality of life outcome. Egger's test indicates no evidence of publication bias for the quality of life outcome (t = -0.43, p = 0.675).


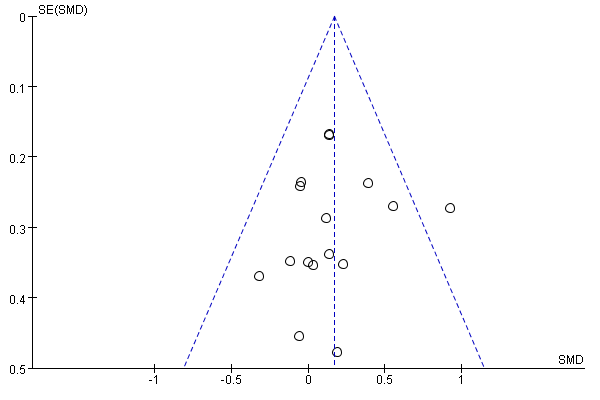


# [Supplementary Table 1](https://www.frontiersin.org/journals/public-health/articles/10.3389/fpubh.2025.1616053/full#SM1)[. PRISMA checklist](#_Table 1. Characteristics of the included studies.)

| **Section and Topic** | **Item #** | **Checklist item** | **Location where item is reported** |
| --- | --- | --- | --- |
| **TITLE** | | |  |
| Title | 1 | Identify the report as a systematic review. | Page 1 |
| **ABSTRACT** | | |  |
| Abstract | 2 | See the PRISMA 2020 for Abstracts checklist. | Pages 1–2 |
| **INTRODUCTION** | | |  |
| Rationale | 3 | Describe the rationale for the review in the context of existing knowledge. | Pages 2–3 |
| Objectives | 4 | Provide an explicit statement of the objective(s) or question(s) the review addresses. | Page 3 |
| **METHODS** | | |  |
| Eligibility criteria | 5 | Specify the inclusion and exclusion criteria for the review and how studies were grouped for the syntheses. | Pages 3–4 |
| Information sources | 6 | Specify all databases, registers, websites, organisations, reference lists and other sources searched or consulted to identify studies. Specify the date when each source was last searched or consulted. | Page 3 |
| Search strategy | 7 | Present the full search strategies for all databases, registers and websites, including any filters and limits used. | [Supplementary Table 2](https://www.frontiersin.org/journals/public-health/articles/10.3389/fpubh.2025.1616053/full#SM1) |
| Selection process | 8 | Specify the methods used to decide whether a study met the inclusion criteria of the review, including how many reviewers screened each record and each report retrieved, whether they worked independently, and if applicable, details of automation tools used in the process. | Page 4 |
| Data collection process | 9 | Specify the methods used to collect data from reports, including how many reviewers collected data from each report, whether they worked independently, any processes for obtaining or confirming data from study investigators, and if applicable, details of automation tools used in the process. | Page 4 |
| Data items | 10a | List and define all outcomes for which data were sought. Specify whether all results that were compatible with each outcome domain in each study were sought (e.g. for all measures, time points, analyses), and if not, the methods used to decide which results to collect. | Page 4 |
|  | 10b | List and define all other variables for which data were sought (e.g. participant and intervention characteristics, funding sources). Describe any assumptions made about any missing or unclear information. | [Supplementary Table 4](https://www.frontiersin.org/journals/public-health/articles/10.3389/fpubh.2025.1616053/full#SM1) |
| Study risk of bias assessment | 11 | Specify the methods used to assess risk of bias in the included studies, including details of the tool(s) used, how many reviewers assessed each study and whether they worked independently, and if applicable, details of automation tools used in the process. | Page 4 |
| Effect measures | 12 | Specify for each outcome the effect measure(s) (e.g. risk ratio, mean difference) used in the synthesis or presentation of results. | Page 4 |
| Synthesis methods | 13a | Describe the processes used to decide which studies were eligible for each synthesis (e.g. tabulating the study intervention characteristics and comparing against the planned groups for each synthesis (item #5)). | [Supplementary Table 4](https://www.frontiersin.org/journals/public-health/articles/10.3389/fpubh.2025.1616053/full#SM1) |
|  | 13b | Describe any methods required to prepare the data for presentation or synthesis, such as handling of missing summary statistics, or data conversions. | Page 4 |
|  | 13c | Describe any methods used to tabulate or visually display results of individual studies and syntheses. | Pages 4–5 |
|  | 13d | Describe any methods used to synthesize results and provide a rationale for the choice(s). If meta-analysis was performed, describe the model(s), method(s) to identify the presence and extent of statistical heterogeneity, and software package(s) used. | Pages 4–5 |
|  | 13e | Describe any methods used to explore possible causes of heterogeneity among study results (e.g. subgroup analysis, meta-regression). | Pages 4–5 |
|  | 13f | Describe any sensitivity analyses conducted to assess robustness of the synthesized results. | Pages 4–5 |
| Reporting bias assessment | 14 | Describe any methods used to assess risk of bias due to missing results in a synthesis (arising from reporting biases). | Page 5 |
| Certainty assessment | 15 | Describe any methods used to assess certainty (or confidence) in the body of evidence for an outcome. | Page 4 |
| **RESULTS** | | |  |
| Study selection | 16a | Describe the results of the search and selection process, from the number of records identified in the search to the number of studies included in the review, ideally using a flow diagram. | Page 5, Figure 1 |
|  | 16b | Cite studies that might appear to meet the inclusion criteria, but which were excluded, and explain why they were excluded. | [Supplementary Table 3](https://www.frontiersin.org/journals/public-health/articles/10.3389/fpubh.2025.1616053/full#SM1) |
| Study characteristics | 17 | Cite each included study and present its characteristics. | [Supplementary Table 4](https://www.frontiersin.org/journals/public-health/articles/10.3389/fpubh.2025.1616053/full#SM1) |
| Risk of bias in studies | 18 | Present assessments of risk of bias for each included study. | Pages 5–6, Figure 2–3 |
| Results of individual studies | 19 | For all outcomes, present, for each study: (a) summary statistics for each group (where appropriate) and (b) an effect estimate and its precision (e.g. confidence/credible interval), ideally using structured tables or plots. | Supplementary Figure 1 |
| Results of syntheses | 20a | For each synthesis, briefly summarise the characteristics and risk of bias among contributing studies. | Pages 6–8 |
|  | 20b | Present results of all statistical syntheses conducted. If meta-analysis was done, present for each the summary estimate and its precision (e.g. confidence/credible interval) and measures of statistical heterogeneity. If comparing groups, describe the direction of the effect. | Pages 6–8 |
|  | 20c | Present results of all investigations of possible causes of heterogeneity among study results. | Pages 6–8, [Supplementary Table 6](https://www.frontiersin.org/journals/public-health/articles/10.3389/fpubh.2025.1616053/full#SM1) |
|  | 20d | Present results of all sensitivity analyses conducted to assess the robustness of the synthesized results. | Pages 6–8, [Supplementary Figure 6](https://www.frontiersin.org/journals/public-health/articles/10.3389/fpubh.2025.1616053/full#SM1) |
| Reporting biases | 21 | Present assessments of risk of bias due to missing results (arising from reporting biases) for each synthesis assessed. | Supplementary Figure 7 |
| Certainty of evidence | 22 | Present assessments of certainty (or confidence) in the body of evidence for each outcome assessed. | [Supplementary Table 5](https://www.frontiersin.org/journals/public-health/articles/10.3389/fpubh.2025.1616053/full#SM1) |
| **DISCUSSION** | | |  |
| Discussion | 23a | Provide a general interpretation of the results in the context of other evidence. | Pages 8–9 |
|  | 23b | Discuss any limitations of the evidence included in the review. | Pages 10–11 |
|  | 23c | Discuss any limitations of the review processes used. | Pages 10–11 |
|  | 23d | Discuss implications of the results for practice, policy, and future research. | Pages 9–10 |
| **OTHER INFORMATION** | | |  |
| Registration and protocol | 24a | Provide registration information for the review, including register name and registration number, or state that the review was not registered. | Page 3 |
|  | 24b | Indicate where the review protocol can be accessed, or state that a protocol was not prepared. | Page 3 |
|  | 24c | Describe and explain any amendments to information provided at registration or in the protocol. |  |
| Support | 25 | Describe sources of financial or non-financial support for the review, and the role of the funders or sponsors in the review. | Page 11 |
| Competing interests | 26 | Declare any competing interests of review authors. | Page 11 |
| Availability of data, code and other materials | 27 | Report which of the following are publicly available and where they can be found: template data collection forms; data extracted from included studies; data used for all analyses; analytic code; any other materials used in the review. | Page 12 |

*From:*  Page MJ, McKenzie JE, Bossuyt PM, Boutron I, Hoffmann TC, Mulrow CD, et al. The PRISMA 2020 statement: an updated guideline for reporting systematic reviews. BMJ 2021;372:n71. doi: 10.1136/bmj.n71. This work is licensed under CC BY 4.0. To view a copy of this license, visit <https://creativecommons.org/licenses/by/4.0/>

# Supplementary Table 2. Search strategies

English database

| **1. Pubmed** | | |  |
| --- | --- | --- | --- |
| #1 | Search:("Digital Health"[Mesh] OR "Digital Technology"[Mesh] OR "Virtual Reality"[Mesh] OR "Augmented Reality"[Mesh] OR "Artificial Intelligence"[Mesh] OR "Telemedicine"[Mesh] OR "Telerehabilitation"[Mesh] OR "Mobile Applications"[Mesh] OR "Exergaming"[Mesh] OR "Videoconferencing"[Mesh]) OR ("Health, Digital"[tiab] OR "Digital Health Technolog*"[tiab] OR eHealth[tiab] OR mHealth[tiab] OR "virtual reality"[tiab] OR VR[tiab] OR "augmented reality"[tiab] OR AR[tiab] OR "Reality, Virtual"[tiab] OR "Augmented Realities"[tiab] OR "Mixed Realit*"[tiab] OR "extended reality"[tiab] OR "artificial intelligence"[tiab] OR AI[tiab] OR "Intelligence, Artificial"[tiab] OR telemedicine[tiab] OR "remote medicine"[tiab] OR "Remote Rehabilitation*"[tiab] OR "Tele rehabilitation*"[tiab] OR "Virtual Rehabilitation*"[tiab] OR "Virtual Medicine"[tiab] OR "Mobile Health"[tiab] OR "Telehealth"[tiab] OR "Videoconference*"[tiab] OR "Video conference*"[tiab] OR "Exergame*"[tiab] OR "Virtual Reality Exercise*"[tiab] OR "Active Video Gaming*"[tiab]) | |  |
| #2 | Search:(("Tai Ji"[Mesh] OR "Qigong"[Mesh] OR "Medicine, Chinese Traditional"[Mesh]) OR (taiji[tiab] OR "tai chi"[tiab] OR "Tai Chi Chuan"[tiab] OR Taijiquan[tiab] OR "T'ai Chi"[tiab] OR "Tai Ji Quan"[tiab] OR qigong[tiab] OR "chi kung"[tiab] OR "Qi Gong"[tiab] OR "Ch'i Kung"[tiab] OR "eight section brocade"[tiab] OR baduanjin[tiab] OR "Eight Pieces of Brocade"[tiab] OR yijinjing[tiab] OR "muscle tendon change"[tiab] OR wuqinxi[tiab] OR "five animal exercise"[tiab] OR "five animal frolics"[tiab] OR daoyin[tiab] OR "Chinese Mind Body Exercise"[tiab] OR "chinese traditional exercise"[tiab])) | |  |
| #3 | #1 OR #2 | |  |
|  | Filters: Randomized Controlled Trial | |  |
| **2. Embase** | | |  |
| #1 | 'digital health'/exp OR 'digital health technology'/exp OR 'virtual reality'/exp OR 'augmented reality'/exp OR 'electronic device'/exp OR 'wearable device'/exp OR 'artificial intelligence'/exp OR 'telemedicine'/exp OR 'telehealth'/exp OR 'video consultation'/exp OR 'telerehabilitation'/exp OR 'mobile application'/exp OR 'exergaming'/exp OR 'videoconferencing'/exp OR 'digital health':ab,ti OR 'virtual reality':ab,ti OR VR:ab,ti OR 'augmented reality':ab,ti OR AR:ab,ti OR 'electronic device':ab,ti OR 'wearable device':ab,ti OR 'artificial intelligence':ab,ti OR 'AI':ab,ti OR eHealth:ab,ti OR mHealth:ab,ti OR 'Mixed Realit*':ab,ti OR 'extended reality':ab,ti OR 'remote medicine':ab,ti OR 'Remote Rehabilitation*':ab,ti OR 'Tele rehabilitation*':ab,ti OR 'Virtual Rehabilitation*':ab,ti OR 'Virtual Medicine':ab,ti OR 'Mobile Health':ab,ti OR 'Active Video Gaming*':ab,ti OR Exergam*:ab,ti | |  |
| #2 | 'Tai Chi'/exp OR 'qigong'/exp OR 'qigong exercise'/exp OR 'baduanjin'/exp OR 'wuqinxi'/exp OR 'yijinjing'/exp OR 'daoyin'/exp OR taiji:ab,ti OR 'Tai Chi':ab,ti OR 'Tai Chi Chuan':ab,ti OR Taijiquan:ab,ti OR 'Tai Ji Quan':ab,ti OR qigong:ab,ti OR 'chi kung':ab,ti OR 'Qi Gong':ab,ti OR 'eight section brocade':ab,ti OR baduanjin:ab,ti OR yijinjing:ab,ti OR wuqinxi:ab,ti OR 'five animal frolics':ab,ti OR daoyin:ab,ti OR 'Chinese Mind Body Exercise':ab,ti OR 'chinese traditional exercise':ab,ti | |  |
| #3 | #1 OR #2 | |  |
| #4 | #3 AND 'randomized controlled trial'/de | |  |
| **3. Cochrane Library** | | |  |
| #1 | MeSH descriptor: [Digital Technology] explode all trees | |  |
| #2 | MeSH descriptor: [Digital Health] explode all trees | |  |
| #3 | (digital NEXT technolog* OR digitization OR smart NEXT technolog* OR "digital health" OR eHealth OR mHealth OR digital NEXT therap* OR "software as medical device"):ti,ab,kw | |  |
| #4 | MeSH descriptor: [Telemedicine] explode all trees | |  |
| #5 | (telemed* OR telehealth OR tele NEXT rehab* OR remote NEXT therap* OR "mobile health" OR "Real time videoconferencing"):ti,ab,kw | |  |
| #6 | MeSH descriptor: [Artificial Intelligence] explode all trees | |  |
| #7 | (AI OR "machine learning" OR "deep learning" OR neural NEXT network* OR "computer vision"):ti,ab,kw | |  |
| #8 | MeSH descriptor: [Virtual Reality] explode all trees | |  |
| #9 | MeSH descriptor: [Exergaming] explode all trees | |  |
| #10 | ("virtual reality" OR VR OR "augmented reality" OR AR OR "mixed reality" OR MR OR "extended reality" OR XR OR exergaming):ti,ab,kw | |  |
| #11 | #1 OR #2 OR #3 OR #4 OR #5 OR #6 OR #7 OR #8 OR #9 OR #10 | |  |
| #12 | MeSH descriptor: [Tai Ji] explode all trees | |  |
| #13 | MeSH descriptor: [Qigong] explode all trees | |  |
| #14 | MeSH descriptor: [Medicine, Chinese Traditional] explode all trees | |  |
| #15 | ("Tai Chi Chuan" OR "Taijiquan" OR "taiji" OR "T'ai Chi" OR "tai ji" OR taichi OR qigong OR "chi kung" OR baduanjin OR "eight section brocade" OR "Eight Sectioned Exercise" OR "Eight Pieces of Brocade" OR yijinjing OR "muscle tendon change" OR wuqinxi OR "five animal frolics" OR "five animal exercise" OR "Five Mimic Animal Boxing" OR daoyin OR "guided exercise" OR "Chinese Mind Body Exercise" OR traditional NEXT chinese NEXT exercise*):ti,ab,kw | |  |
| #16 | #12 OR #13 OR #14 OR #15 | |  |
| #17 | #11 AND #16 | |  |
| #18 | MeSH descriptor: [Randomized Controlled Trials as Topic] explode all trees | |  |
| #19 | (randomized OR RCT OR "controlled trial" OR placebo OR sham):ti,ab,kw | |  |
| #20 | #18 OR #19 | |  |
| #21 | #17 AND #20 | |  |
| **4. Web of Science** | | | |
| #1 | | TS=("Digital Health" OR "Digital Technology" OR "Virtual Reality" OR "Augmented Reality" OR "Artificial Intelligence" OR "Telemedicine" OR "Telerehabilitation" OR "Mobile Applications" OR "Exergaming" OR "Videoconferencing" OR "Health, Digital" OR "Digital Health Technolog*" OR eHealth OR mHealth OR VR OR AR OR "Reality, Virtual" OR "Augmented Realities" OR "Mixed Realit*" OR "extended reality" OR AI OR "Intelligence, Artificial" OR telemedicine OR "remote medicine" OR "Remote Rehabilitation*" OR "Tele rehabilitation*" OR "Virtual Rehabilitation*" OR "Virtual Medicine" OR "Mobile Health" OR "Telehealth" OR "Videoconference*" OR "Video conference*" OR "Exergame*" OR "Virtual Reality Exercise*" OR "Active Video Gaming*" OR "wearable device*" OR "Health, Digital") | |
| #2 | | TS=("Tai Ji" OR "Qigong" OR "Medicine, Chinese Traditional" OR taiji OR "tai chi" OR "Tai Chi Chuan" OR Taijiquan OR "T'ai Chi" OR "Tai Ji Quan" OR qigong OR "chi kung" OR "Qi Gong" OR "Ch'i Kung" OR "eight section brocade" OR baduanjin OR "Eight Pieces of Brocade" OR yijinjing OR "muscle tendon change" OR wuqinxi OR "five animal exercise" OR "five animal frolics" OR daoyin OR "Chinese Mind Body Exercise" OR "chinese traditional exercise") | |
| #3 | | #1 AND #2 | |

Chinese Databas

| **5. China National Knowledge Infrastructure（CNKI）** | | | |  |  |
| --- | --- | --- | --- | --- | --- |
| #1 | (SU=('数字化技术' + '数字健康' + '数字疗法' + '虚拟现实' + '增强现实' + '人工智能' + '远程医学' + '远程康复' + '体感游戏' + '移动应用') OR TKA=('数字化技术' + '数字健康' + '数字疗法' + '虚拟现实' + '增强现实' + '混合现实' + '扩展现实' + '人工智能' + 'VR' + 'AR' + 'AI' + 'MR' + 'XR' + '远程医学' + '远程诊疗' + '远程健康' + '远程治疗' + '实时视频' + '可穿戴设备' + '体感识别' + '动作捕捉' + '体感游戏')) | | |  |  |
| #2 | (SU=('太极' + '气功' + '八段锦' + '易筋经' + '五禽戏' + '导引术' + '医学, 中国传统') OR TKA=('太极' + '气功' + '八段锦' + '易筋经' + '五禽戏' + '导引术' + '中医身心训练' + '中医功法' + '呼吸导引' + '中医传统功法' + '中医传统运动' + '中医传统武术')) | | |  |  |
| #3 | #1 AND #2 | | |  |  |
| **6. Wanfang med online** | | | | |  |
| #1 | | (主题:(“数字健康” OR “数字化技术” OR “虚拟现实” OR “增强现实” OR “人工智能” OR “远程医学” OR “远程康复” OR “移动应用” OR “体感游戏”) OR 标题:(“数字健康” OR “数字化技术” OR “数字疗法” OR “虚拟现实” OR "VR" OR “增强现实” OR “AR” OR “混合现实” OR “MR” OR “扩展现实” OR “XR” OR “人工智能” OR “AI” OR “远程医学” OR “远程康复” OR “远程医疗” OR “移动健康” OR “移动应用” OR “体感互动” OR “可穿戴设备” OR “体感识别”) OR 关键词:(“数字健康” OR “数字化技术” OR “数字疗法” OR “虚拟现实” OR "VR" OR “增强现实” OR “AR” OR “混合现实” OR “MR” OR “扩展现实” OR “XR” OR “人工智能” OR “AI” OR “远程医学” OR “远程康复” OR “远程医疗” OR “移动健康” OR “移动应用” OR “体感互动” OR “可穿戴设备” OR “体感识别”)) | | |  |
| #2 | | (主题:(“太极” OR “气功” OR “八段锦” OR “易筋经” OR “五禽戏” OR “医学, 中国传统”) OR 标题:(“太极” OR “气功” OR “八段锦” OR “易筋经” OR “五禽戏” OR “导引术” OR “导引功法” OR “中医传统功法” OR “中医传统运动”) OR 关键词:(“太极” OR “气功” OR “八段锦” OR “易筋经” OR “五禽戏” OR “导引术” OR “导引功法” OR “中医传统功法” OR “中医传统运动”)) | | |  |
| #3 | | #1 AND #2 | | |  |
| **7. VIP Database** | | | | | |
| #1 | | | (M=(“数字健康” OR “数字化技术” OR “数字疗法” OR “虚拟现实” OR "VR" OR “增强现实” OR “AR” OR “混合现实” OR “MR” OR “扩展现实” OR “XR” OR “人工智能” OR “AI” OR “远程医学” OR “远程康复” OR “远程医疗” OR “移动健康” OR “移动应用” OR “运动游戏” OR “体感游戏” OR “体感互动” OR “可穿戴设备” OR “体感识别”) OR R=(“数字健康” OR “数字化技术” OR “数字疗法” OR “虚拟现实” OR "VR" OR “增强现实” OR “AR” OR “混合现实” OR “MR” OR “扩展现实” OR “XR” OR “人工智能” OR “AI” OR “远程医学” OR “远程康复” OR “远程医疗” OR “移动健康” OR “移动应用” OR “体感游戏” OR “运动游戏” OR “体感互动” OR “可穿戴设备” OR “体感识别”)) | | |
| #2 | | | (M=(“太极” OR “气功” OR “八段锦” OR “易筋经” OR “五禽戏” OR “导引术” OR “导引功法” OR “中医传统功法” OR “中医传统运动”) OR R=(“太极” OR “气功” OR “八段锦” OR “易筋经” OR “五禽戏” OR “导引术” OR “导引功法” OR “中医传统功法” OR “中医传统运动”)) | | |
| #3 | | | #1 AND #2 | | |

# [Supplementary Table 3](https://www.frontiersin.org/journals/public-health/articles/10.3389/fpubh.2025.1616053/full#SM1). Studies excluded by checking the full-text articles

| **Ineligible population (n = 5)** |
| --- |
| [1] Gunes Gencer GY, Cetin SY, Kara DS, Yardim S, Ayan A. The effects of baduanjin qigong exercise via telerehabilitation in ankylosing spondylitis: A randomized controlled study. Explore (NY). 2025;21(2):103078. doi:10.1016/j.explore.2024.103078 |
| [2] AKINCI, Buket, et al. Feasibility and safety of Qigong training delivered from two different digital platforms in physically inactive adults: a pilot randomized controlled study. Eur J Integr Med, 2022, 54: 102171. [https://doi.org/10.1016/j.eujim.2022.102171](https://doi.org/10.1016/j.eujim.2022.102171) |
| [3] 许清,纪美芳,程宏,等.远程康复指导下八段锦锻炼次数与COVID-19患者心肺功能的相关性分析[J].卫生职业教育,2021,39(16):139-141.DOI:CNKI:SUN:ZDYX.0.2021-16-068. |
| [4] Gao Z, Ryu S, Chen Y. Effects of Tai Chi App and Facebook health education programs on breast cancer survivors' stress and quality of life in the Era of pandemic. Complement Ther Clin Pract. 2022;48:101621. doi:10.1016/j.ctcp.2022.101621 |
| [5] 周宇璇,李三军,周云英,等.基于虚拟现实技术的中医传统功法对扩张型心肌病患者心功能及生活质量影响的研究[J].中国医学创新,2024,21(06):90-94.DOI:CNKI:SUN:ZYCX.0.2024-06-022. |
| **Ineligible intervention (n = 17)** |
| [1] Guo H, Cao J, He S, et al. Quantifying the Enhancement of Sarcopenic Skeletal Muscle Preservation Through a Hybrid Exercise Program: Randomized Controlled Trial. JMIR Aging. 2024;7:e58175. doi:10.2196/58175 |
| [2] He S, Wei M, Meng D, Wang Z, Yang G, Wang Z. Self-determined sequence exercise program for elderly with sarcopenia: A Randomized controlled trial with clinical assistance from explainable artificial intelligence. Arch Gerontol Geriatr. 2024;119:105317. doi:10.1016/j.archger.2023.105317 |
| [3] DIETER, Valerie, et al. EVALUATION OF A 12-WEEK APP-GUIDED EXERCISE INTERVENTION IN PATIENTS WITH KNEE OSTEOARTHRITIS (RE. FLEX): RESULTS OF A RANDOMIZED CONTROLLED TRIAL. Osteoarthritis and Cartilage, 2024, 32: S234-S235. |
| [4] Franke M., Weenink M., Hegeman M., Gerritsen D., Bode C., Vonkeman H.E. (2024). HPR THE FEASIBILITY OF VIRTUAL REALITY SUPPORTED EXERCISE THERAPY FOR AXIAL SPONDYLOARTHRITIS IN A HOME AND A PHYSIOTHERAPY SETTING: A MIXED METHODS STUDY FROM A MULTI-DISCIPLINARY PERSPECTIVE, European Congress of Rheumatology, EULAR 2024. Annals of the Rheumatic Diseases. https://dx-doi-org.webvpn.cams.cn/10.1136/annrheumdis-2024-eular.2187 |
| [5] Garcia LM, Birckhead BJ, Krishnamurthy P, et al. An 8-Week Self-Administered At-Home Behavioral Skills-Based Virtual Reality Program for Chronic Low Back Pain: Double-Blind, Randomized, Placebo-Controlled Trial Conducted During COVID-19. J Med Internet Res. 2021;23(2):e26292. doi:10.2196/26292 |
| [6] Provan SA, Litleskare S, Flaten OE, Pettersen H, Røset L, Calogiuri G. Participatory Development of a Virtual Reality Exercise Program for People with Chronic Pain. Games Health J. 2024;13(5):332-334. doi:10.1089/g4h.2023.0229 |
| [7] Haolin T, Yuanbin Y, Hu Z, et al. Efficacy of Daoyin combined with lower limb robot as a comprehensive rehabilitation intervention for stroke patients: a randomized controlled trial. J Tradit Chin Med. 2024;44(3):530-536. doi:10.19852/j.cnki.jtcm.20240322.002 |
| [8] 徐渝,胡盼.体感游戏联合八段锦对脑卒中患者应用价值的研究[J].现代医药卫生,2024,40(03):391-394.DOI:CNKI:SUN:XYWS.0.2024-03-007. |
| [9] 周倩,曹峰,蒋紫娟,等.Switch体感游戏联合五禽戏在帕金森病患者功能训练中的应用效果研究[J].当代护士(上旬刊),2023,30(10):134-137.DOI:10.19791/j.cnki.1006-6411.2023.28.036. |
| [10] 贾晖.体感游戏联合五禽戏训练对脑梗死患者心理状态、肢体运动功能、生活质量的影响[J].中外医学研究,2023,21(14):123-127.DOI:10.14033/j.cnki.cfmr.2023.14.031. |
| [11] 张晓羽,赵海滨.八段锦结合虚拟康复训练系统在老年稳定型心绞痛运动康复中的应用[J].环球中医药,2018,11(08):1233-1237.DOI:CNKI:SUN:HQZY.0.2018-08-015. |
| [12] Chen SC, Lin CH, Su SW, Chang YT, Lai CH. Feasibility and effect of interactive telerehabilitation on balance in individuals with chronic stroke: a pilot study. J Neuroeng Rehabil. 2021;18(1):71. doi:10.1186/s12984-021-00866-8 |
| [13] 孙志成,王彤,顾晓美,马金霖,张娜,董亚军,郭睿,钰李慧.虚拟现实训练对养老机构主观认知下降患者认知功能和功能性体适能的影响[J].中华物理医学与康复杂志,2023,45(4):320-325.DOI:10.3760/cma.j.issn.0254-1424.2023.04.007. |
| [14] Liao YY, Chen IH, Hsu WC, Tseng HY, Wang RY. Effect of exergaming versus combined exercise on cognitive function and brain activation in frail older adults: A randomised controlled trial. Ann Phys Rehabil Med. 2021;64(5):101492. doi:10.1016/j.rehab.2021.101492 |
| [15] Liao YY, Chen IH, Wang RY. Effects of Kinect-based exergaming on frailty status and physical performance in prefrail and frail elderly: A randomized controlled trial. Sci Rep. 2019;9(1):9353. doi:10.1038/s41598-019-45767-y |
| [16] Adcock M, Fankhauser M, Post J, et al. Effects of an In-home Multicomponent Exergame Training on Physical Functions, Cognition, and Brain Volume of Older Adults: A Randomized Controlled Trial. Front Med (Lausanne). 2020;6:321. Published 2020 Jan 28. doi:10.3389/fmed.2019.00321 |
| [17] 黄嘉滢,马子霖,程天翊,等.可穿戴设备干预下八段锦对老年心衰患者的临床疗效观察[J].中国老年保健医学,2024,22(01):12-16.DOI:CNKI:SUN:LNBJ.0.2024-01-003. |
| **Ineligible study design (n = 5)** |
| [1] Yin Z, Martinez CE, Li S, et al. Adapting Chinese Qigong Mind-Body Exercise for Healthy Aging in Older Community-Dwelling Low-income Latino Adults: Pilot Feasibility Study. JMIR Aging. 2021;4(4):e29188. Published 2021 Nov 1. doi:10.2196/29188 |
| [2] Du Y, Patel N, Hernandez A, et al. Examining the Delivery of a Tailored Chinese Mind-Body Exercise to Low-Income Community-Dwelling Older Latino Individuals for Healthy Aging: Feasibility and Acceptability Study. JMIR Form Res. 2022;6(9):e40046. Published 2022 Sep 13. doi:10.2196/40046 |
| [3] Adcock M, Sonder F, Schättin A, Gennaro F, de Bruin ED. A usability study of a multicomponent video game-based training for older adults. Eur Rev Aging Phys Act. 2020;17:3. Published 2020 Jan 11. doi:10.1186/s11556-019-0233-2 |
| [4] Adcock M, Thalmann M, Schättin A, Gennaro F, de Bruin ED. A Pilot Study of an In-Home Multicomponent Exergame Training for Older Adults: Feasibility, Usability and Pre-Post Evaluation. Front Aging Neurosci. 2019;11:304. Published 2019 Nov 22. doi:10.3389/fnagi.2019.00304 |
| [5] Hsieh CC, Lin PS, Hsu WC, et al. The Effectiveness of a Virtual Reality-Based Tai Chi Exercise on Cognitive and Physical Function in Older Adults with Cognitive Impairment. Dement Geriatr Cogn Disord. 2018;46(5-6):358-370. doi:10.1159/000494659 |
| **Insufficient data (n=2)** |
| [1] Yang Y, McCluskey S, Bydon M, et al. A Tai chi and qigong mind-body program for low back pain: A virtually delivered randomized control trial. N Am Spine Soc J. 2024;20:100557. Published 2024 Sep 8. doi:10.1016/j.xnsj.2024.100557 |
| [2] Wu G, Keyes L, Callas P, Ren X, Bookchin B. Comparison of telecommunication, community, and home-based Tai Chi exercise programs on compliance and effectiveness in elders at risk for falls. Arch Phys Med Rehabil. 2010;91(6):849-856. doi:10.1016/j.apmr.2010.01.024 |

# [Supplementary Table 4](https://www.frontiersin.org/journals/public-health/articles/10.3389/fpubh.2025.1616053/full#SM1). Characteristics of the included studies

| Study | Country | Study design | Age (Years) | Participant characteristics | Sample size | Experimental group (EG) | Control group (CG) | Time, Frequency, Duration | Outcomes |
| --- | --- | --- | --- | --- | --- | --- | --- | --- | --- |
| Chen PJ [34]. 2020 | China | RCT | EG (72.2 ± 2.8), TCEs (75.1 ± 5.5) | Community-dwelling older adults | 28 (EG: 14; TCEs: 14) | EG: Tai Chi assisted by augmented reality | TCEs : Tai Chi | 30 min/time, thrice per week, 8 weeks | Functional Mobility: TUG Balance: BBS |
| Chen XS [35]. 2025 | China | RCT | EG (66 ± 5), ST (65 ±  8), TAU (64 ± 7) | Older adults with type 2 diabetes and mild cognitive impairment | 162 (EG: 54; ST: 54; TAU: 54) | EG: Home-based online Tai Chi training | ST: fitness walking TAU: treatment as usual | 30 min/time, thrice per week, 12 weeks | Global Cognition: MoCA Quality of life: SF-36 |
| He S [36]. 2024 | China | RCT | EG1 (73.67 ± 4.77), EG2 (72.26 ± 4.43), TCEs (70.91 ± 3.94) | Sarcopenic older adults | 70 (EG1: 24; EG2: 23; TCEs: 23) | EG1: AI-enhanced remote Tai Chi training EG2: Home-based online Tai Chi training | TCEs: Tai Chi | 40 min/time, thrice per week, 12 weeks | Functional Mobility: TUG Grip: Hand Dynamometer Quality of life: SF-36 |
| Li F [37]. 2023 | United States | RCT | EG1 (76.0 ± 5.1), EG2 (75.9 ± 5.1), ST (76.0 ± 6.1) | Community-dwelling older adults with mild cognitive impairment | 318 (EG1: 105; EG2: 107; ST: 106) | EG1: Home-based online cognitively enhanced Tai Chi training EG2: Home-based online Tai Chi training | ST: Home-based online stretching training | 60 min/time, twice per week, 24 weeks | Functional Mobility: TUG Balance: 4SBT Global Cognition: MoCA Depression: GDS-15 Quality of life: EQ-5D |
| Li F [38]. 2022 | United States | RCT | EG1 (74.4 ± 5.1), EG2 (74.5 ± 5.6), ST (74.9 ± 6.3) | Community-dwelling older adults with mild cognitive impairment | 69 (EG1: 23; EG2: 22; ST: 24) | EG1: Home-based online cognitively enhanced Tai Chi training EG2: Home-based online Traditional Tai Chi training | ST: Home-based online stretching training | 60 min/time, twice per week, 16 weeks | Global Cognition: MoCA |
| Li F [39]. 2021 | United States | RCT | EG (76.1 ± 6.2), ST (76.2 ± 6.3) | Community-dwelling older adults with mild cognitive impairment | 30 (EG: 15; ST: 15) | EG: Home-based online cognitively enhanced Tai Chi training | ST: Home-based online stretching training | 60 min/time, twice per week, 24 weeks | Functional Mobility: TUG Balance: 4SBT |
| Lo HHM [40]. 2025 | China | RCT | EG (65.7 ± 5.5), TCEs (64.6 ± 5.7) | Older adults with chronic musculoskeletal pain | 69 (EG: 33; TCEs: 36) | EG: Tai Chi assisted by virtual reality | TCEs: Tai Chi | 60 min/time, once per week, 8 weeks | Depression: PHQ-9 Quality of life: EQ-5D |
| Liu CL [41]. 2022 | China | RCT | EG (74.6 ± 6.1), TCEs (73.2 ±  6.3), TAU (73.4 ± 6.5) | Older adults with mild cognitive impairment | 50 (EG: 16; TCEs: 17; TAU: 17) | EG: Exergaming-based Tai Chi | TCEs: Tai Chi TAU: treatment as usual | 50 min/time, thrice per week, 12 weeks | Global Cognition: MoCA |
| Li X [42]. 2024 | China | RCT | EG (65.2 ± 4.2); TCEs (64.9 ±  3.7) | Community-dwelling older adults | 50 (EG: 21; TCEs: 29) | EG: AI-enhanced Tai Chi | TCEs: Tai Chi | 40 min/time, thrice per week, 8 weeks | Balance: OLS Grip: Hand Dynamometer Depression: BDI Quality of life: SF-12 |
| Meng D [43]. 2025 | China | RCT | EG1 (71.15 ± 7.95), EG2 (69.41 ± 7.34), TCEs (70.77 ±  8.57) | Sarcopenic older adults | 78 (EG1: 26; EG2: 27; TCEs: 25) | EG1: AI-enhanced remote Ba Duan Jin training EG2: Home-based online Ba Duan Jin training | TCEs: Ba Duan Jin | 40 min/time, thrice per week, 12 weeks | Functional Mobility: TUG Grip: Hand Dynamometer Quality of life: SF-36 |
| Qiu T [44]. 2024 | China | RCT | Average age 62 years | Older adults with depression | 300 (EG: 100; TCEs: 100; TAU: 100) | EG: Tai Chi assisted by virtual reality | TCEs: Tai Chi TAU: treatment as usual | once per week, 24 weeks | Depression: GDS-30 |
| Sun ZC [45]. 2020 | China | RCT | EG ( 73.96 ± 6.32);TAU ( 72.53 ±  7.04) | Older adults with osteoporosis in old people’s home | 60 (EG: 30; TAU: 30) | EG: Ba Duan Jin assisted by virtual reality | TAU: treatment as usual | 50 min/time, thrice per week, 48 weeks | Quality of life: SF-36 |
| Sun ZC [46]. 2021 | China | RCT | Range 65-85 | Older adults with mild cognitive impairment in old people’s home | 57 (EG: 29; TAU: 28) | EG: Ba Duan Jin assisted by virtual reality | TAU: treatment as usual | 50 min/time, thrice per week, 24 weeks | Global Cognition: MoCA Quality of life: QOL-AD |
| Wei M [47]. 2025 | China | RCT | EG1 (71.57 ± 7.24), EG2 (72.56 ± 7.76), TCEs (70.77 ±  8.27) | Sarcopenic older adults | 76 (EG1: 27; EG2: 25; TCEs: 24) | EG1: AI-enhanced remote Yi Jin Jing training EG2: Home-based online Yi Jin Jing training | TCEs: Yi Jin Jing | 40 min/time, thrice per week, 12 weeks | Functional Mobility: TUG Grip: Hand Dynamometer Quality of life: SF-36 |
| Zhang XY [48]. 2024 | China | RCT | Range 60-80 | Community-dwelling older adults with mild cognitive impairment | 145 (EG: 50; TCEs: 48; TAU: 47) | EG: Ba Duan Jin assisted by augmented reality | TCEs: Ba Duan Jin TAU: treatment as usual | 60 min/time, thrice per week, 24 weeks | Global Cognition: MoCA |
| Zhang Y [49]. 2024 | China | RCT | ≥ 60 | Community-dwelling older adults | 42 (EG: 14; TCEs1: 16; TCEs2: 12) | EG: AI-enhanced Tai Chi | TCEs1: Tai Chi with coach's movement guidance TCEs2: Tai Chi without coach's movement guidance | 40 min/time, thrice per week, 8 weeks | Balance: OLS Grip: Hand Dynamometer Depression: BDI Quality of life: SF-12 |
| RCT: randomized controlled trial. ST: sham therapy. TCEs: traditional Chinese exercises. TAU: treatment as usual. AI: artificial intelligence. TUG: Timed Up and Go. BBS: Berg Balance Scale. SF-36: Short-Form 36-Item Health Survey. 4SBT: 4-Stage Balance Test. MoCA: Montreal Cognitive Assessment. 15-GDS: 15-item Geriatric Depression Scale. EQ-5D: Self-report European quality of life-5 dimensions. PHQ-9: Patient Health Questionnaire-9. OLS: One-Leg Stance. BDI: Beck Depression Inventory. SF-12: Short-Form 12-Item Health Survey. 30-GDS: 30-item Geriatric Depression Score. QOL-AD: Quality of Life-Alzheimer's Disease. | | | | | | | | | |

# [Supplementary Table 5](https://www.frontiersin.org/journals/public-health/articles/10.3389/fpubh.2025.1616053/full#SM1). Certainty of evidence using GRADE

| Outcomes | Study Design | Risk of Bias | Inconsistency | Indirectness | Imprecision | Publication Bias | Number of Studies | Numbers of Participants | WMD/SMD (95% CI) | Certainty |
| --- | --- | --- | --- | --- | --- | --- | --- | --- | --- | --- |
| Functional Mobility | RCTs | Seriousa | Seriousb | Not serious | Not serious | Not serious | 10 | 600 | WMD = -0.81 [-1.28, -0.33] | Low |
| Balance Function | RCTs | Seriousa | Not serious | Not serious | Not serious | Not serious | 7 | 468 | SMD = 0.88 [0.68, 1.08] | Moderate |
| Grip Strength | RCTs | Seriousa | Not serious | Not serious | Seriousc | Not serious | 9 | 316 | WMD = -0.26 [-1.15, 0.62] | Low |
| Cognition Function | RCTs | Seriousa | Seriousb | Not serious | Not serious | Not serious | 11 | 801 | WMD = 1.98 [1.44, 2.52] | Low |
| Depression | RCTs | Seriousa | Seriousb | Not serious | Seriousc | Not serious | 8 | 779 | SMD = -0.71 [-1.48, 0.05] | Very low |
| Quality of Life | RCTs | Seriousa | Not serious | Not serious | Not serious | Not serious | 16 | 982 | SMD = 0.17 [0.03, 0.30] | Moderate |
| WMD: weighted mean differences. SMD: standard mean difference. RCTs: randomized controlled trials. aDowngrade due to the low methodological quality: The design of the trial has a large bias in randomization, allocation concealment, or blinding. bDowngrade due to large heterogeneity: I2 statistics >50%, which can be reasonably explained by prior assumptions. cDowngrade due to imprecision: the confidence interval for the pooled estimate includes both potential benefit and harm. | | | | | | | | | | |

# [Supplementary Table 6](https://www.frontiersin.org/journals/public-health/articles/10.3389/fpubh.2025.1616053/full#SM1). Results of the analysis of individual outcome indicators and their subgroups

| Physical function | | | | | | | | |
| --- | --- | --- | --- | --- | --- | --- | --- | --- |
| Outcomes |  |  |  | Number of comparisons | Number of participants | WMD/SMD (95% CI) | P value | I² (%) |
|  | | | | | | | | |
| Functional Mobility |  |  | Total | 10 | 600 | WMD = -0.81 [-1.28, -0.33] | p = 0.0009 | 76% |
|  | Control type | | Nonspecific active (ST) | 3 | 348 | WMD = -1.62 [-2.14, -1.11] | p < 0.00001 | 51% |
|  |  |  | Specific active (TCEs) | 7 | 252 | WMD = -0.40 [-0.76, -0.03] | p = 0.03 | 30% |
|  | Subgroup difference: p = 0.0001 | | |  |  |  |  |  |
|  | Total duration | | < 24 weeks | 7 | 252 | WMD = -0.40 [-0.76, -0.03] | p = 0.03 | 30% |
|  |  |  | ≥ 24 weeks | 3 | 348 | WMD = -1.62 [-2.14, -1.11] | p < 0.00001 | 51% |
|  | Subgroup difference: p = 0.0001 | | |  |  |  |  |  |
|  | Session duration | | ≤ 40 min | 7 | 252 | WMD = -0.40 [-0.76, -0.03] | p = 0.03 | 30% |
|  |  |  | > 40 min | 3 | 348 | WMD = -1.62 [-2.14, -1.11] | p < 0.00001 | 51% |
|  | Subgroup difference: p = 0.0001 | | |  |  |  |  |  |
|  | Frequency | | Twice per week | 3 | 348 | WMD = -1.62 [-2.14, -1.11] | p < 0.00001 | 51% |
|  |  |  | Thrice per week | 7 | 252 | WMD = -0.40 [-0.76, -0.03] | p = 0.03 | 30% |
|  | Subgroup difference: p = 0.0001 | | |  |  |  |  |  |
|  | | | | | | | | |
| Balance |  |  | Total | 7 | 468 | SMD = 0.88 [0.68, 1.08] | p < 0.00001 | 0% |
|  | Control type | | Nonspecific active (ST) | 3 | 348 | SMD = 0.85 [0.62, 1.08] | p < 0.00001 | 0% |
|  |  |  | Specific active (TCEs) | 4 | 120 | SMD = 0.98 [0.59, 1.38] | p < 0.00001 | 0% |
|  | Subgroup difference: p = 0.56 | | |  |  |  |  |  |
|  | Total duration | | < 24 weeks | 4 | 120 | SMD = 0.98 [0.59, 1.38] | p < 0.00001 | 0% |
|  |  |  | ≥ 24 weeks | 3 | 348 | SMD = 0.85 [0.62, 1.08] | p < 0.00001 | 0% |
|  | Subgroup difference: p = 0.56 | | |  |  |  |  |  |
|  | Session duration | | ≤ 40 min | 4 | 120 | SMD = 0.98 [0.59, 1.38] | p < 0.00001 | 0% |
|  |  |  | > 40 min | 3 | 348 | SMD = 0.85 [0.62, 1.08] | p < 0.00001 | 0% |
|  | Subgroup difference: p = 0.56 | | |  |  |  |  |  |
|  | Frequency | | Twice per week | 3 | 348 | SMD = 0.85 [0.62, 1.08] | p < 0.00001 | 0% |
|  |  |  | Thrice per week | 4 | 120 | SMD = 0.98 [0.59, 1.38] | p < 0.00001 | 0% |
|  | Subgroup difference: p = 0.56 | | |  |  |  |  |  |
|  | | | | | | | | |
| Grip Strength |  |  | Total | 9 | 316 | WMD = -0.26 [-1.15, 0.62] | p = 0.56 | 0% |
|  | | | | | | | | |
| Cognition function | | | | | | | | |
| Outcomes |  |  |  | Number of comparisons | Number of participants | WMD/SMD (95% CI) | P value | I² (%) |
|  | | | | | | | | |
| Global Cognition |  |  | Total | 11 | 801 | WMD = 1.98 [1.44, 2.52] | p < 0.00001 | 84% |
|  | Control type | | Inactive (TAU) | 4 | 235 | WMD = 2.70 [1.26, 4.15] | p = 0.0002 | 87% |
|  |  |  | Nonspecific active (ST) | 5 | 468 | WMD = 1.77 [0.96, 2.59] | p < 0.0001 | 76% |
|  |  |  | Specific active (TCEs) | 2 | 98 | WMD = 1.14 [0.84, 1.45] | p < 0.00001 | 0% |
|  | Subgroup difference: p = 0.05 | | |  |  |  |  |  |
|  | Total duration | | < 24 weeks | 6 | 281 | WMD = 1.41 [0.98, 1.85] | p < 0.00001 | 52% |
|  |  |  | ≥ 24 weeks | 5 | 520 | WMD = 2.70 [1.70, 3.71] | p < 0.00001 | 87% |
|  | Subgroup difference: p = 0.02 | | |  |  |  |  |  |
|  | Session duration | | ≤ 40 min | 2 | 162 | WMD = 0.88 [-0.36, 2.12] | p = 0.16 | 0% |
|  |  |  | > 40 min | 9 | 639 | WMD = 2.12 [1.54, 2.71] | p < 0.00001 | 87% |
|  | Subgroup difference: p = 0.08 | | |  |  |  |  |  |
|  | Frequency | | Twice per week | 4 | 387 | WMD = 1.89 [1.01, 2.77] | p < 0.0001 | 80% |
|  |  |  | Thrice per week | 7 | 414 | WMD = 2.05 [1.31, 2.79] | p < 0.00001 | 86% |
|  | Subgroup difference: p = 0.79 | | |  |  |  |  |  |
|  | | | | | | | | |
| Mental health | | | | | | | | |
| Outcomes |  |  |  | Number of comparisons | Number of participants | WMD/SMD (95% CI) | P value | I² (%) |
|  | | | | | | | | |
| Depression |  |  | Total | 8 | 779 | SMD = -0.71 [-1.48, 0.05] | p = 0.07 | 95% |
|  | Control type | | Inactive (TAU) | 1 | 150 | SMD = -3.83 [-4.38, -3.27] | p < 0.00001 | NA |
|  |  |  | Nonspecific active (ST) | 2 | 318 | SMD = -0.53 [-0.77, -0.29] | p < 0.0001 | 0% |
|  |  |  | Specific active (TCEs) | 5 | 311 | SMD = -0.19 [-0.67, 0.29] | p = 0.44 | 70% |
|  | Subgroup difference: p < 0.00001 | | |  |  |  |  |  |
|  | Total duration | | < 24 weeks | 4 | 161 | SMD = 0.02 [-0.32, 0.36] | p = 0.92 | 10% |
|  |  |  | ≥ 24 weeks | 4 | 618 | SMD = -1.39 [-2.57, -0.21] | p = 0.02 | 97% |
|  | Subgroup difference: p = 0.02 | | |  |  |  |  |  |
|  | Frequency | | Once per week | 3 | 369 | SMD = -1.43 [-3.53, 0.67] | p = 0.18 | 98% |
|  |  |  | Twice per week | 2 | 318 | SMD = -0.53 [-0.77, -0.29] | p < 0.0001 | 0% |
|  |  |  | Thrice per week | 3 | 92 | SMD = -0.17 [-0.60, 0.25] | p = 0.42 | 0% |
|  | Subgroup difference: p = 0.24 | | |  |  |  |  |  |
|  | | | | | | | | |
| Quality of life | | | | | | | | |
| Outcomes |  |  |  | Number of comparisons | Number of participants | WMD/SMD (95% CI) | P value | I² (%) |
|  | | | | | | | | |
| Quality of life |  |  | Total | 16 | 982 | SMD = 0.17 [0.03, 0.30] | p = 0.01 | 3% |
|  | Control type | | Inactive (TAU) | 3 | 198 | SMD = 0.60 [0.31, 0.89] | p < 0.0001 | 9% |
|  |  |  | Nonspecific active (ST) | 3 | 399 | SMD = 0.10 [-0.11, 0.31] | p = 0.35 | 0% |
|  |  |  | Specific active (TCEs) | 10 | 385 | SMD = 0.01 [-0.20, 0.22] | p = 0.91 | 0% |
|  | Subgroup difference: p = 0.004 | | |  |  |  |  |  |
|  | Total duration | | < 24 weeks | 12 | 547 | SMD = 0.06 [-0.12, 0.23] | p = 0.52 | 0% |
|  |  |  | ≥ 24 weeks | 4 | 435 | SMD = 0.30 [0.10, 0.50] | p = 0.003 | 63% |
|  | Subgroup difference: p = 0.07 | | |  |  |  |  |  |
|  | Session duration | | ≤ 40 min | 11 | 478 | SMD = 0.08 [-0.11, 0.27] | p = 0.43 | 0% |
|  |  |  | > 40 min | 5 | 504 | SMD = 0.25 [0.07, 0.43] | p = 0.008 | 59% |
|  | Subgroup difference: p = 0.20 | | |  |  |  |  |  |
|  | Frequency | | Once per week | 1 | 69 | SMD = -0.05 [-0.53, 0.42] | p = 0.82 | NA |
|  |  |  | Twice per week | 2 | 318 | SMD = 0.14 [-0.10, 0.37] | p = 0.26 | 0% |
|  |  |  | Thrice per week | 13 | 595 | SMD = 0.21 [0.04, 0.38] | p = 0.01 | 16% |
|  | Subgroup difference: p = 0.56 | | |  |  |  |  |  |
| WMD: weighted mean differences. SMD: standard mean difference. TAU: treatment as usual. ST: sham therapy. TCEs: traditional Chinese exercises. | | | | | | | | |
